# Allosteric communication between ligand binding domains modulates substrate inhibition in adenylate kinase

**DOI:** 10.1073/pnas.2219855120

**Published:** 2023-04-24

**Authors:** David Scheerer, Bharat V. Adkar, Sanchari Bhattacharyya, Dorit Levy, Marija Iljina, Inbal Riven, Orly Dym, Gilad Haran, Eugene I. Shakhnovich

**Affiliations:** ^a^Department of Chemical and Biological Physics, Weizmann Institute of Science, Rehovot 761001, Israel; ^b^Department of Chemistry and Chemical Biology, Harvard University, Cambridge, MA 02138

**Keywords:** single-molecule FRET, enzymatic activity, protein dynamics

## Abstract

Enzymes play a vital role in life processes; they control chemical reactions and allow functional cycles to be synchronized. Many enzymes harness large-scale motions of their domains to achieve tremendous catalytic prowess and high selectivity for specific substrates. One outstanding example is provided by the three-domain enzyme adenylate kinase (AK), which catalyzes phosphotransfer between ATP to AMP. Here we study the phenomenon of substrate inhibition by AMP and its correlation with domain motions. Using single-molecule FRET spectroscopy, we show that AMP does not block access to the ATP binding site, neither by competitive binding to the ATP cognate site nor by directly closing the LID domain. Instead, inhibitory concentrations of AMP lead to a faster and more cooperative domain closure by ATP, leading in turn to an increased population of the closed state. The effect of AMP binding can be modulated through mutations throughout the structure of the enzyme, as shown by the screening of an extensive AK mutant library. The mutation of multiple conserved residues reduces substrate inhibition, suggesting that substrate inhibition is an evolutionary well conserved feature in AK. Combining these insights, we developed a model that explains the complex activity of AK, particularly substrate inhibition, based on the experimentally observed opening and closing rates. Notably, the model indicates that the catalytic power is affected by the microsecond balance between the open and closed states of the enzyme. Our findings highlight the crucial role of protein motions in enzymatic activity.

## Significance

How conformational dynamics affect the catalytic activity of enzymes remains a topic of active debate. We focus here on the domain closure dynamics of adenylate kinase (AK) and how they are affected by substrate inhibition. By screening an extensive mutant library, we show that this feature of the enzyme is well conserved in evolution. Importantly, domain closure is required in order to bring AK’s substrates to close together for their chemical reaction; single-molecule FRET studies directly measure the populations of the open and closed states. We find that overpopulation of the closed state can be detrimental to activity. The results allow us to develop a kinetic model that properly accounts for AK kinetics by combining conformational dynamics and biochemical steps.

**Fig. 1.**
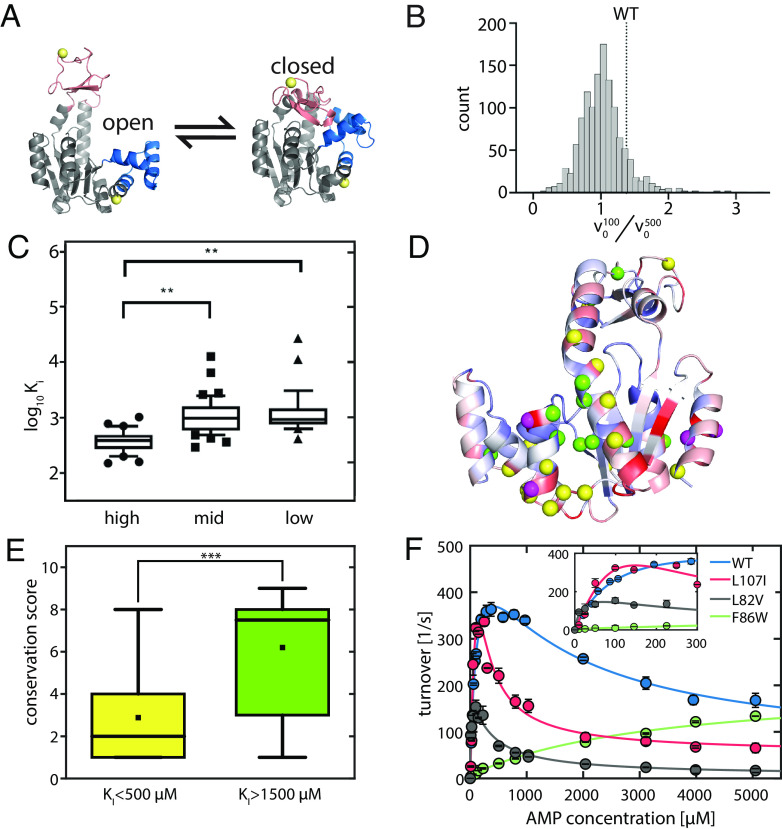
Structural and kinetic attributes of AK variants. (*A*) Structure of AK with its three domains. The CORE domain (gray) connects the LID domain (pink) and the NMP domain (blue). The yellow spheres indicate the positions for the attachment of fluorescent dyes. The protein can undergo a conformational change from the open state (*left*, PDB 4AKE) towards the closed conformation (*right*, PDB 1AKE). (*B*) Distribution of in-lysate activity of 1,248 colonies in terms of the screening parameter $v_{0}^{100}$ / $v_{0}^{500}$ . WT corresponds to a $v_{0}^{100}$ / $v_{0}^{500}$ = 1.38. (*C*) Distribution of *K_I_* values of purified proteins from library variants with high (*top* 64), mid (64 around median) and low (*Bottom* 64) values of $v_{0}^{100}$ / $v_{0}^{500}$ measured in-lysate. (*D*) Conservation score mapped onto the open state with high to low conservation represented as blue to red. The positions with mutants having a *K_I_* ≤ 500 μM for AMP are shown as yellow spheres, while those with *K_I_* ≥ 1,500 μM are shown in green. The four magenta spheres are positions where both inhibited and uninhibited mutations are observed (see *SI Appendix*, Table S1). The conservation scores were obtained from the ConSurf web server (24, 25). (*E*) The distributions of the conservation scores in the strong and weak inhibition bins were found to be statistically different (*P* < 0.01, Student’s *t* test). The mutations that result in strong inhibition (*K_I_* ≤ 500) usually occur at less conserved positions (2.9 ± 0.6), while inhibition-relieving mutations (*K_I_* ≥ 1,500) occur mostly at highly conserved positions (6.2 ± 0.9). (*F*) Activity curves of WT AK and selected mutants show increased SI (L107I, L82V) or loss of inhibition (F86W).

Enzymes have been designed by nature to accelerate vital chemical reactions by many orders of magnitude. Quite a few of them have evolved to harness large-scale motions of domains and subunits to promote their activity. The study of the structural dynamics of these proteins is essential to decipher how they function and are regulated, as has been demonstrated in multiple experimental and theoretical reports (1–4). The enzyme adenylate kinase (AK) is a paradigmatic example of a strong relation between conformational dynamics and catalytic activity (5–8). AK plays a key role in maintaining ATP levels in cells by catalyzing the reaction ATP + AMP ⇄ ADP + ADP (9, 10). It consists of three domains: the large CORE domain, the LID domain that binds ATP, and the nucleotide monophosphate (NMP)-binding domain that binds AMP. X-ray crystallographic studies showed that the LID and NMP domains undergo a major conformational rearrangement upon substrate binding (Fig. 1*A*) (11–13). This movement, termed domain closure, forms the enzyme’s active center and helps to exclude solvent molecules that might interfere with the chemical reaction. AK’s domain-closure dynamics have been studied using NMR spectroscopy (6–8, 14) and fluorescence experiments (5, 6, 15), as well as multiple molecular dynamics simulations (16–21). In particular, our recent single-molecule FRET (smFRET) experiments demonstrated that AK’s opening and closing rates are significantly faster than previously reported (22). In the presence of substrates, domain closure is completed in just a few tens of microseconds. These are likely the fastest large-scale conformational dynamics measured to date, and they are orders of magnitude faster than the overall turnover rate of the enzyme. It was proposed that these fast domain movements might assist the enzyme in orienting its substrates optimally for catalysis. This hypothesis was recently supported by molecular dynamics simulations (23), which clearly demonstrated the effect of domain motions on the orientation of bound substrates.

Interestingly, AK also incurs a pronounced AMP-mediated substrate inhibition (SI) (26, 27). Despite many efforts, the mechanism behind SI has not been elucidated convincingly. While some studies have hinted toward the competitive binding of AMP at the ATP site (28), others claimed that AMP substrate inhibition in AK is uncompetitive in relation to ATP (26). Whether large-scale domain motions in AK relate to SI has also been debated. Bulk FRET studies have indicated that inhibition arises due to substantial closing of the LID domain upon AMP binding (27). However, it was unclear whether this effect was induced by direct binding of AMP to the ATP site (competitive inhibition at the ATP site) or through allosteric modulation (i.e., AMP binding at its cognate site affects LID domain movement). Other studies suggested that at high AMP concentrations, binding of AMP before the release of ADP from the LID domain might lead to the formation of an abortive complex that causes inhibition (26). The latter effect could arise if LID opening is slow and is the rate-limiting step in catalysis, as has been proposed (6, 14, 15).

In this work, we carried out experimental and computational studies on several inhibition-prone as well as noninhibited mutants of AK (29) to determine the mechanism of AMP-mediated SI in AK. Based on the screening of an extensive AK mutant library, we show that mutations throughout the enzyme’s structure modulate SI, suggesting that it has been under positive selection during evolution. We establish that inhibition cannot be explained by either competitive binding of AMP at the ATP site or spurious LID domain closure by AMP in the absence of ATP. Instead, we find that for inhibition-prone variants of AK, AMP facilitates domain closure at lower concentrations of ATP, which may interfere with proper substrate binding mechanics required for the reaction. Based on these results, we propose a kinetic model that combines conformational dynamics and chemical steps to explain the enzymatic activity of AK with substrate inhibition.

## Results

### Structural and Kinetic Attributes of AK Variants with Substrate Inhibition.

In an earlier study (29), we characterized a series of mutants of AK and found a correlation between AMP-mediated SI and protein stability. An important question that arises is whether there is a specific region that is responsible for the gain or loss of SI, possibly by affecting how the substrates bind to the protein. To answer this, we created an extensive library of AK mutants and characterized their SI profiles. We particularly sought to identify mutations that alter SI without perturbing the enzymatic activity. Accordingly, using the crystal structure of AK with the bound inhibitor Ap_5_A (12), we first identified 71 residues that were at least 8 Å away from the inhibitor and their sidechains were not involved in any interactions with the rest of the protein (*SI Appendix*, Fig. S1*A*, see details in *Methods*). We then generated a library of 923 mutants wherein 13 different amino acids were introduced at each of these 71 locations identified above (*Methods*). Next, we developed a screening method (*SI Appendix*, Fig. S1*B*) to measure the extent of AMP-mediated SI directly from the cell lysate of 1248 individual colonies by measuring the ratio of the initial reaction velocity at two different concentrations of AMP (100 μM and 500 μM, Fig. 1*B*).

We selected ~200 candidates from this dataset that showed lower, higher, and similar SI levels compared to the WT protein and sequenced them by Sanger sequencing to recover 86 unique mutations with clean chromatograms in the first pass. We further purified these proteins and measured their activity profiles (Fig. 1*C*). For a quantitative comparison between the mutants and literature values, the AMP-related Michaelis constant (*K_M_*) and inhibition constant (*K_I_*) were obtained by a fit to a model of uncompetitive inhibition (*Methods*), and the values are given in *SI Appendix*, Table S1. We mapped the positions of the inhibition-prone mutants (*K_I_* ≤ 500) and the noninhibited mutants (*K_I_* ≥ 1,500) on the structure of AK (Fig. 1*D*). Both effects can be observed for residues distributed all over the structure rather than in specific regions of the protein. As shown in Fig. 1*E*, we observed that at those locations where mutation led to a loss of inhibition, the WT residue had a statistically significant (*P* < 0.01, Student’s *t* test) higher conservation than those that led to an increase of inhibition. [The conservation score was determined by the frequency of occurrence in a multiple sequence alignment (24, 25)]. In other words, the gain of SI upon mutation tends to occur at positions of low conservation, while the loss of SI occurs at highly conserved locations. This signifies that SI in AK has been under positive selection during evolution. Since back-to-consensus mutations are generally stabilizing (30), this plot also signifies that higher protein stability generally leads to more SI in AK, as seen from our previous study (29).

The mutant screening experiment did not identify a specific structural attribute that is responsible for SI by AMP. Further, a crystallographic study of one of the mutants also did not demonstrate a structural change that may explain the effect of AMP. In particular, we determined the crystal structure of L107I AK, which shows strong inhibition, in complex with Ap_5_A to a resolution of 2.05 Å. The structure was found to be very similar to other closed AK structures (12, 31, 32) (*SI Appendix*, Fig. S2), with a 0.3 Å RMSD from the 1AKE structure (12). As no differences in the static structure of AK were found that could explain differences in enzymatic activity, these are more likely rooted in dynamic interactions. Therefore, we turned to studies of the dynamic properties of AK mutants in relation to SI.

### From Biochemistry to Structural Dynamics.

To explain the origins of SI in AK and similar multisubstrate enzymes, previous studies have primarily considered three hypotheses. The first involved binding of the inhibiting substrate to an additional site (e.g., the ATP site) (33). The second proposed uncompetitive inhibition by AMP through blocking the accessibility of the ATP binding site (26, 27). Finally, it was suggested that kinetic differences between alternating pathways towards the fully substrate-bound complex could lead to inhibition (34).

To probe the origins of SI further, we selected three variants of AK with similar thermal stabilities to the WT: L82V and L107I (29), which are more inhibited than the WT, and F86W, which is not inhibited by AMP (26). Michaelis–Menten curves for these variants as a function of AMP concentration are shown in Fig. 1*F*. We first investigated if SI in AK arises due to AMP having a second binding site in addition to the one on the NMP domain. The additional binding site could be the ATP-binding site—as AMP is a close structural analog of ATP—or any other yet-unknown site. In that context, we found that for the WT (*SI Appendix*, Fig. S3*A* and Table S2), the presence of excess ATP led to a gradual and dose-dependent loss in SI, which is a classic apparent sign of competitive inhibition at the ATP binding site. In other words, it seemed possible that the competitive binding of AMP at the ATP binding site at high concentrations causes inhibition, which is relieved by excess ATP in the solution. If this were true, then under conditions when the AMP concentration vastly surpasses the ATP concentration, binding of AMP to the ATP site would be preferred and result in a complete loss of activity. Contrary to this expectation, the enzyme retains significant residual activity even at high AMP concentrations (5, 26, 27), as seen particularly well for L107I (Fig. 1*F* and *SI Appendix*, Fig. S3*B*).

**Fig. 2.**
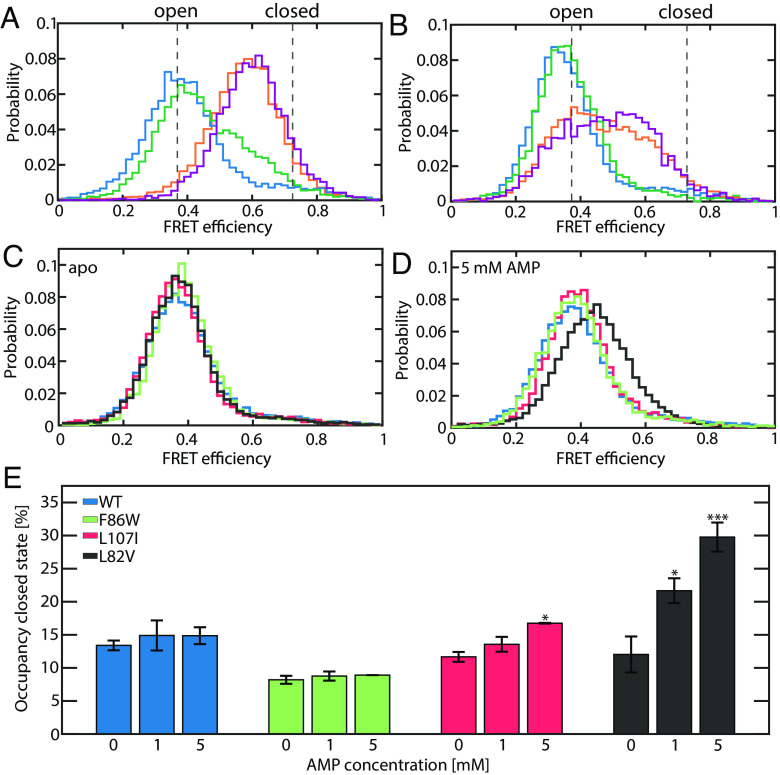
AMP does not close the LID domain. (*A* and *B*) FRET efficiency histograms of the WT protein. (*A*) The apoprotein (blue trace) mainly adopts an open conformation, occasionally exploring the closed state. The binding of ATP increases the population of the closed state, as demonstrated by histograms at 100 μM (green) and 1 mM (purple) ATP. When 1 mM AMP is added, a significant population of the closed state is attained even at a low ATP concentration of 50 μM (orange). In this experiment, ADP was also added at concentrations that maintained the equilibrium of the enzymatic reaction (*Methods*). The dashed lines indicate the most likely positions of the open and closed state (0.37/0.72 for the WT, respectively). (*B*) AMP alone, even at 30 mM (green), leads to very minimal changes in FRET efficiency compared to the apoprotein (blue). ATP-γ-S, a slowly hydrolyzable analog of ATP, binds to the ATP binding site and triggers partial closure at 50 μM (orange). In the presence of both ligands (purple), ATP-γ-S is still bound to the ATP-binding site and is not outcompeted by AMP, which would trigger LID opening. Instead, the binding of AMP to its own site even promotes closure to a minor extent. (*C*) For the apoprotein, the FRET efficiency histogram of the WT (blue) and all mutants are similar. Shown are F86W (green), L107 (red) and L82V (black). (*D*) FRET efficiency histogram for all mutants in the presence of 5 mM AMP. Only for L82V a significant shift to higher FRET efficiency is detected. (*E*) Values for the occupancy of the closed state as a function of AMP concentration were obtained by H^2^MM analysis. The experiment confirms that the occupancy of the closed state for the WT (blue), L107I (red) and F86W (green) is not significantly altered by AMP alone. In contrast, a partial closure is observed for L82V (black). Asterisks indicate the significance of the deviation of parameters from the apoprotein (***: *P* < 0.01, **: *P* < 0.05, *: *P* < 0.1, no index: *P* > 0.1, Student’s *t* test). Error bars are given as the SEM based on three independently prepared samples.

We therefore asked: Does AMP inhibit AK by binding to the ATP binding site in the LID domain, or does it affect the catalytic properties differently? To gain insight into this question, we turned to smFRET spectroscopy to measure the domain movements directly. AK was labeled at residues 73 and 142, positioned in the CORE and LID domains, respectively (Fig. 1*A*), and studied in solution (22). In the absence of substrates, AK molecules yielded a FRET efficiency histogram dominated by a peak at ~0.4 (Fig. 2*A*, blue), corresponding to the open conformation. A shoulder towards higher FRET efficiency values indicated that the closed conformation is also sampled, though to a small extent (6, 22, 35). The addition of ATP (Fig. 2*A*, green and purple) shifted the histogram towards higher FRET efficiency values, resulting from a considerable increase in the population of the closed conformation. In the presence of 1 mM AMP (orange), similar shifts were observed, but they required much lower concentrations of ATP.

Surprisingly, in the presence of high concentrations of AMP as a sole substrate, the FRET efficiency histogram of the WT protein remained unchanged (Fig. 2*B*), indicating that AMP alone cannot cause LID domain closure. To see whether the effect of AMP is different in mutants with stronger or weaker SI, we also double-labeled and measured the three variants introduced above (L107I, L82V, and F86W). None of these single-point mutants significantly affected the FRET efficiency histogram of the apoenzyme (Fig. 2*C*), and the addition of AMP alone did not induce a significant shift (Fig. 2*D*). The only exception was L82V, the most inhibited mutant, which showed a slight shift toward higher FRET efficiencies. To accurately retrieve changes in the populations of the open and closed conformations of the enzyme under various conditions, we used the hidden Markov model-based analysis method H^2^MM (36). This algorithm employs photon arrival times from single-molecule signals as input for an optimization process that obtains both occupancies and rates of exchange of conformational states and is capable of retrieving microsecond dynamics (22). Representative trajectories including state assignments using the Viterbi algorithm are shown in *SI Appendix*, Fig. S4. The model we used involved only two conformational states, open and closed (Fig. 1*A*). The resulting parameters were validated with different tests as outlined in the *SI Appendix*, Figs. S5–S8 and Tables S3 and S4. A notable increase in the population of the closed state by AMP was seen only for L82V (Fig. 2*E*). Interestingly, both closing (*SI Appendix*, Fig. S9*A*) and opening (*SI Appendix*, Fig. S9*B*) rates were accelerated in the binary AK-AMP complex. This effect was particularly substantial for L107I and L82V. In contrast, no changes were seen for the uninhibited F86W.

Clearly, AMP alone does not promote the closed state of the LID domain in AK. However, this does not rule out that AMP binds to the ATP site and blocks access for the native substrate, thereby inhibiting the protein. To check this point, we tested whether AMP can competitively replace ATP from its cognate binding site. To limit the potential effect of ADP, which binds to both the ATP and AMP site, we utilized the slowly hydrolyzable ATP analog ATP-γ-S. In the presence of 50 μM ATP-γ-S, partial closure was observed (Fig. 2*B*). If AMP can displace ATP/ATP-γ-S from its native binding site, adding a vast excess of AMP should induce a shift towards the open conformation. Instead, we observed a minor shift towards the closed state in the FRET efficiency histograms, rendering the possibility that AMP acts as a competitive substrate inhibitor very unlikely.

To provide support to the point that AMP does not bind at the ATP site, in addition to the single-molecule studies, we rationally designed a mutant Q92Y that is predicted to weaken AMP binding at its cognate site, based on the structure of *Escherichia coli* AK with bound Ap_5_A (12). The mutation indeed led to a loss in activity for both the WT (*SI Appendix*, Fig. S10*A*) and L82V (*SI Appendix*, Fig. S10*B*). Using Differential Scanning Fluorimetry to obtain the change in thermal melting temperatures of AK upon ligand binding as a function of ligand concentration, we found that Q92Y on the background of WT weakens AMP binding (*SI Appendix*, Fig. S10*C*). On the background of the highly inhibited mutant L82V (L82V-Q92Y mutant), it completely abolished AMP binding (*SI Appendix*, Fig. S10*D*), while ATP binding was retained for both WT and L82V backgrounds (*SI Appendix*, Fig. S10 *E* and *F*). This experiment indicated that AMP cannot bind anywhere but to its cognate site. This was also confirmed by isothermal titration calorimetry studies, which found no more than one binding site for both ATP and AMP (37).

### AMP Binding Has an Allosteric Effect on the LID Domain.

**Fig. 3.**
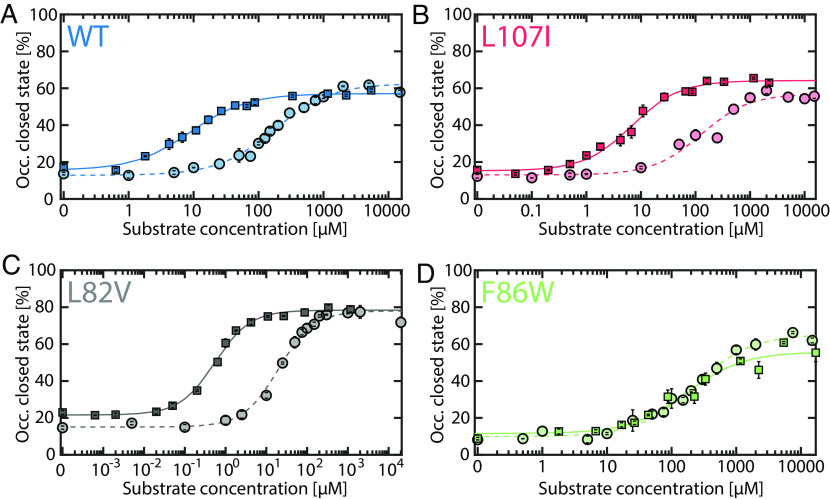
ATP-dependent closure of the LID domain. The occupancy of the closed state was determined by H^2^MM in the presence of either solely ATP (circles) or also 1 mM AMP and appropriate concentrations of ADP to maintain equilibrium, as described in *Methods* (squares). In all variants, the LID domain closes at high ATP levels. For (*A*) WT, (*B*) L107I and (*C*) L82V, the presence of 1 mM AMP promotes closure at much lower ATP concentrations. In the noninhibited mutant (*D*) F86W, AMP has no significant effect. Dashed (ATP only) and straight (all substrates) lines indicate fits to a binding model as described in *SI Appendix*, *Supplementary Note 1*. Error bars are given as the SEM based on two to three independently prepared samples. As in Fig. 2*A*, ADP was added to maintain equilibrium; the substrate concentration on the abscissa is the total concentration of ligands for the ATP binding site (i.e., ATP + ADP, *SI Appendix*, Table S5).

Given the observed effect of AMP binding at its own site on LID domain dynamics, it is imperative to find whether AMP further modulates the effect of ATP on domain closure. Fig. 3 shows how the population of the closed state of the enzyme changes in response to substrate binding to the LID domain, either with ATP alone or also in the presence of 1 mM AMP. (In the latter case, ADP was also added to maintain the enzymatic reaction under equilibrium- see *Methods* and *SI Appendix*, Table S5). The very presence of ATP as a sole substrate (Fig. 3, circles) enabled a large conformational change from a predominantly open to a more closed conformation. The maximum occupancy that could be achieved for the closed state was 56-78%, in agreement with previous studies (22, 38). Fitting the curves in Fig. 3 to simple binding isotherms provided the transition midpoint concentrations (which we call *C*_50_), which were found to correlate with the strength of substrate inhibition (*SI Appendix*, Table S6). The impact of a fixed concentration of AMP on the ATP-dependent population of the closed state is shown as squares in Fig. 3. LID domain closure occurred at a 20 to 40 times lower substrate concentration in the presence of AMP (*SI Appendix*, Table S6), indicating remarkable cooperativity in substrate binding. This cooperativity was almost entirely absent for the non-inhibited mutant F86W (Fig. 3*D*). Interestingly, previous work has shown that AMP binding reduces the *K_M_* for ATP in the WT but not in the F86W mutant (26). The strongest cooperativity was observed for the most inhibited mutant, L82V (Fig. 3*C*). Furthermore, in this mutant, the closed state was significantly more populated (78.4 ± 0.7%) than in the WT (57.1 ± 0.6%).

**Fig. 4.**
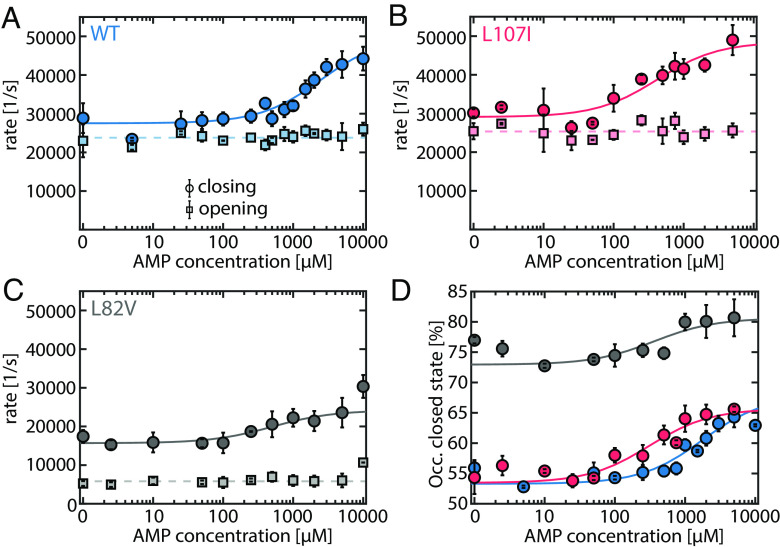
AMP accelerates LID domain closing. (*A*–*C*) Opening (circles, solid lines) and closing (squares, dashed lines) rates for (*A*) the WT (blue), (*B*) L107I (red) and (*C*) L82V (gray) in the presence of increasing concentrations of AMP and a fixed concentration of ATP (1 mM). Opening rates are not affected by the presence of AMP. The closing rates were fitted to a model described in *SI Appendix*, *Supplementary Note 1*. (*D*) Occupancy of the closed state based on the rates shown in (*A*–*C*). Error bars are given as the SEM based on two to three independently prepared samples.

It is clear from the results of Fig. 3 that AMP has an effect on the closure of the LID domain, even though it does not directly bind to that domain. AMP must therefore affect the LID domain allosterically. To shed further light on the effect of AMP on LID domain mechanics, we studied domain-closure dynamics at a fixed concentration of ATP (1 mM, together with specified concentrations of ADP to maintain equilibrium) and increased concentrations of AMP (Fig. 4).

As reported earlier (22), both opening and closing rates are much faster than the enzymatic turnover (*SI Appendix*, Table S7). In the WT protein in the absence of AMP (Fig. 4*A*), closing rates (27,307 ± 752 s^−1^) were slightly faster than opening rates (23,937 ± 1,123 s^−1^). Strikingly, the gradual addition of AMP did not affect the rate of domain opening. As for the closing rate, small AMP concentrations—at which no inhibition is detected (up to ~1 mM)—also did not have a measurable impact. However, at inhibitory AMP concentrations (>1 mM), domain closure was significantly accelerated (up to 50,289 ± 2,795 s^−1^), leading to a higher occupancy of the closed state of 63.5 ± 0.4% (Fig. 4*D*). Interestingly, this overpopulation of the closed state coincided with the range of AMP concentrations where the most substantial drop in the activity (Fig. 1*F*) was observed. A very similar picture was seen with the more inhibited variants (Fig. 4 *B* and *C*). No measurable impact of AMP binding was detected for the opening rates, whereas the closing rates increased above 100 μM. A *C_50_* value of 2,568 ± 820 μM was found for the WT, with significantly lower values (~500 μM) for L107I and L82V. Moreover, L107I was shifted further toward the closed state (64.8 ± 0.5%) than it was possible in the presence of ATP alone (55.8 ± 0.8%). For L82V, the population of the closed state (75.5 ± 0.8%) was the largest, which might match that mutant’s overall low activity. No enhancement of the closing rate was detected for the noninhibited mutant F86W (similar to *SI Appendix*, Fig. S9).

### A Model to Explain Substrate Inhibition.

**Fig. 5.**
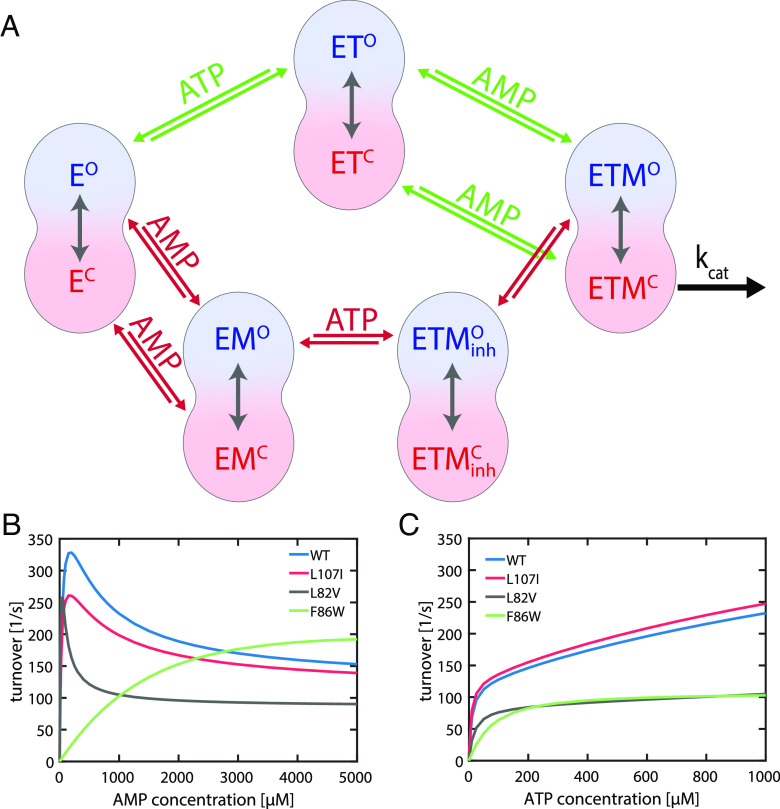
A model to explain substrate inhibition. (*A*) Scheme of the model, which is based on two competing pathways: When ATP binds first (green pathway) to the apoenzyme (E), this results in the ATP-bound state ET. Subsequent binding of AMP leads to a productive closed state (ETM) that undergoes the chemical step of the enzyme in the closed conformation (ETM^C^). However, if AMP binds first (red pathway, via EM), subsequent binding of ATP causes the formation of ${ETM}_{inh}$ , which deviates from the productive state ETM in the orientation of the substrates. It is assumed that the closed state ${\text{ETM}}_{\text{inh}}^{C}$ does not enable phosphotransfer due to an inappropriate substrate orientation. The corresponding open conformation ${ETM}_{inh}^{O}$ must convert to ETM^O^ to enable catalysis. Binding or reorientation of ATP, which lead to a productive catalytic conformation, cannot occur in the closed conformation (23). (*B* and *C*) Simulated activity curves of WT and mutants as a function of (*B*) AMP and (*C*) ATP concentrations based on the model (*A*) and experimentally observed parameters of opening and closing rates. The rate of conversion from ETM^O^ to ${ETM}_{inh}^{O}$ was fixed at 250 s^−1^. The model shows AMP-mediated SI for WT, L107I and L82V, but not for F86W. No SI was observed as a function of ATP concentration, in accordance with experimental data.

Given that AMP inhibits AK’s enzymatic activity and also affects domain-closure dynamics, we postulated that the two phenomena are correlated. Therefore, we devised a kinetic model for AK, based on the hypothesis that different closed states can be sampled, depending upon the order of ligand binding. Some of these states are incompatible with catalysis and must convert to the catalysis-prone state before the reaction occurs. As per our model (Fig. 5*A*), the apoenzyme E can be present in both open (E^O^, blue) and closed (E^C^, red) states. (States are defined only for the ATP-binding LID domain.) The status of the LID domain does not affect the binding of AMP to the NMP domain; hence, both open and closed states can bind AMP. However, ATP can bind only to the open state.

If ATP binds first at the LID domain, ET^O^ and ET^C^, i.e., open and closed conformations of the ATP-bound protein, respectively, are formed. Both of these states can subsequently bind AMP and form a double substrate-bound state, ETM. If AMP binds first at the NMP domain to form EM^O^ and EM^C^, the consequent binding of ATP leads to the formation of ${ETM}_{inh}$ . ETM and ${ETM}_{inh}$ are distinguished by their binding order—ATP first, then AMP or vice versa—based on a hypothesis that initial AMP binding may result in a limited or different sampling of the relative orientation of the nucleotides (see below). Hence, ${ETM}_{inh}^{C}$ is an unproductive closed state, while ETM^C^ is productive. The two ETM states can interconvert only through their open states, as suggested recently by our MD simulations (23). At the same time, phosphoryl transfer can happen only through ETM^C^. In essence, we have introduced a kinetic hindrance for the path that binds AMP first. When a typical activity assay of AK is carried out in the bulk in the presence of saturating ATP concentrations, at low AMP levels a greater flux of the reaction goes through the pathway involving the productive ETM intermediate. However, as AMP levels increase, a larger fraction of the total flux goes through the pathway involving the unproductive ${ETM}_{inh}$ state, leading to a drop in the activity.

To simulate kinetics based on this model, we used experimentally observed opening and closing rates of the LID domain for E (E^O^ ⇌ E^C^), ET (ET^O^ ⇌ ET^C^), EM (EM^O^ ⇌ EM^C^), ETM (ETM^O^ ⇌ ETM^C^) as input in the model (*SI Appendix*, Table S8). We assumed that each species has one specific opening and closing rate. The concentration of nucleotides determined the occupancy of each of these states (22). The single-molecule experiments could not distinguish between ET and ETM, as no change in the apparent protein dynamics was observed upon the addition of AMP at noninhibiting concentrations (Fig. 4). Therefore, the rates adopted for these two states were identical. The rates for the ${ETM}_{inh}$ state ( ${ETM}_{inh}^{O}$ ⇌ ${ETM}_{inh}^{C}$ ) were derived from asymptotic values obtained from the binding isotherm fits of Fig. 4 (*SI Appendix*, *Supplementary Note 1*). We also assumed a single rate constant for the actual phosphotransfer step (500 s^−1^) and for the conversion rate between ${ETM}_{inh}^{O}$ and ETM^O^ (250 s^−1^) for all mutants. Nucleotide binding rates are given in *SI Appendix*, Table S9.

Under these model assumptions, a simulation of the enzyme activity at a saturating ATP concentration (1 mM) and varying AMP concentrations (Fig. 5*B*) was able to qualitatively reproduce the SI pattern of the WT as well as the mutants L107I and L82V. Further, no inhibition was observed for the F86W mutant, as expected. At the same time, using a saturating AMP concentration (1 mM) and varying ATP concentrations, the simulation did not show SI for any mutant (Fig. 5*C*). The ability to obtain the correct level of inhibition by AMP for the different mutants provides a strong support for the model. While in principle we could use the model to quantitatively fit the enzyme activity curves, we believe that our lack of knowledge on some of the involved parameters does not currently warrant such a calculation. Future studies might shed light on several of the unknown parameters, facilitating a more quantitative analysis.

We used the model to obtain some further insight into the role of domain closure dynamics in enzyme activity. To this end, we altered the rates of LID domain closing of each of the ETM states in turn, using one of two ways (*SI Appendix*, Fig. S11): either by preserving the open/closed ratio *K*_C_ or by preserving the opening rate, similar to the experimentally observed effect of AMP. In both scenarios, all other parameters were kept constant. As long as the equilibrium ratio *K*_C_ was preserved (*SI Appendix*, Fig. S11*A*), varying the closing rate involving ${ETM}_{inh}^{O}$ did not affect activity. With *K*_C_ preserved, also varying the rate involving ETM^O^ had a minor effect (*SI Appendix*, Fig. S11*B*). A much more substantial impact of varying the closing rate involving ${ETM}_{inh}^{O}$ was observed when the opening rate was fixed (*SI Appendix*, Fig. S11*C*). When the conformational equilibrium was altered, the unproductive states became more populated, which caused stronger inhibition. In contrast, for the productive closing (*SI Appendix*, Fig. S11*D*), enhancing the domain closure rate had a positive effect on turnover.

An important criterion determining SI strength is the conversion rate between ${ETM}_{inh}^{O}$ and ETM^O^. *SI Appendix*, Fig. S11*E* depicts how an increase in this rate can almost completely thwart SI while a reduction in the rate increases the effect. In our simulations (Fig. 5 *B* and *C*), we assumed a constant interconversion rate for all mutants, 250 s^−1^. In reality, the potent inhibition observed experimentally for L107I and L82V might also be influenced by a slower transition between ${ETM}_{inh}^{O}$ and ETM^O^ for these mutants and also by other parameters whose accurate values cannot be determined currently.

## Discussion

Despite its comparatively small size, AK’s ubiquity and central role in cellular metabolism have attracted numerous studies. In particular, the complex interplay between conformational changes and enzymatic activity has been frequently investigated. An important aspect here is the AMP-mediated SI, which has been studied for several decades, but no mechanism has been conclusively established. In a previous work, several stabilizing and destabilizing point mutations were introduced into AK, and the mutants exhibited many extremes of SI (from very strong to no SI at all) (29). In the present work, we used several of those mutants (covering a range of SI behavior) to pinpoint a mechanism for inhibition. We show that neither uncompetitive nor competitive binding of AMP can explain SI in AK. Instead, we find that inhibition arises due to strong allosteric communication between the NMP and LID domains, which manifests in a highly cooperative closing of the LID domain for inhibition-prone mutants in the presence of both ligands (ATP and AMP) as compared to ATP alone. Based on this, we propose a model for the enzymatic activity that explains why greater sampling of the LID closed state in the presence of AMP and ATP might lead to SI. According to this model, the order of substrate binding matters. In particular, the initial binding of AMP followed by ATP leads to a closed state that does not allow the correct positioning of the ligands for effective phosphate transfer. Using the experimentally observed opening and closing rates for each state of the enzyme, this model can effectively reproduce the SI profiles of the different mutants studied. The enzymatic cycle of AK has traditionally been understood as a random bi–bi mechanism where the two substrates ATP and AMP can bind in any order. Our data show that AK activity follows a particular class of random reactions: the substrates can bind in any order, but the outcome of the reaction is different along the two pathways.

Among the two inhibition-prone mutants, L82V displayed somewhat different properties than L107I. Being the most inhibition prone, L82V showed an increase in closed-state occupancy with AMP alone, indicating that the allosteric communication between the LID and NMP sites is the highest in this mutant. Nevertheless, even for this mutant, opening rates are higher than closing rates in the presence of only AMP; hence, structural inaccessibility of the ATP binding site cannot be the reason for SI. In the presence of both AMP and ATP, the closed state occupancy for L82V is the highest among all mutants (78% vs. 63 to 65% for others), which is likely the reason for the potent inhibition and overall low activity. In contrast, F86W displayed minimal impact of AMP on LID domain dynamics, and likewise, no inhibition.

Controlling the equilibrium between the open and closed state in AK provides a way to regulate enzymatic activity. Varying this conformational equilibrium by other means, like mutations or osmolytes, was shown to affect enzymatic catalysis directly (22, 39). In this context, we found that urea can lift AMP-mediated inhibition by repopulating the open state. We will focus on this effect in a future study.

The maximal catalytic rate of AK is attained at or near the expected physiological concentration of AMP [~300 μM (39–41)]. Changes in AMP concentration will accordingly lead to changes in AK’s activity, which can substantially affect other processes in the cell by altering the availability of nucleotides. For example, we have recently shown that elevated AMP concentrations decrease the fitness of *E.coli* cells expressing inhibition-prone AK strains (29). Paalme et al. demonstrated that AMP levels in *E.coli* cells depend on which carbon sources are available; for example, AMP levels are elevated when only acetate is present as a carbon source (42). For mitochondrial AK, it was shown that phosphorylation of AMP to ADP is inhibited when AMP levels sharply increase [for example during ischemia (43)], thus reducing the amount of adenine nucleotides available for oxidative phosphorylation (44). The current work, based on an extensive library of AK mutants and subsequent informatics analyses, shows that residues favoring substrate inhibition are often well conserved, suggesting that substrate inhibition in AK has a high functional value. Our recent study of mutant *E.coli* AK strains shows the highest bacterial growth rates for AK variants with a *K_I_* similar to the WT (29); *E.coli* AK might have evolved to represent the ideal tradeoff between efficient AMP binding and thermal stability on one side and SI on the other side (29). Even single-point mutations can change this balance, as our library data clearly show. Interestingly, SI can be modulated through mutations that span most regions of the proteins, suggesting that long-range allosteric communication is a general feature of AK.

It is important to mention that previous work on AK dynamics suggested that the LID opening rate is the rate-determining step in catalysis (14, 15). In contrast, a recent study demonstrated an enhanced tendency toward enzyme opening for several mutants with a low catalytic activity, suggesting that domain opening is not rate-limiting at least in those mutants (45). Our smFRET data in this study and the previous one (22) demonstrate that rates of domain movement (both opening and closing) are orders of magnitude faster than the enzymatic turnover, which implies that the enzyme opens and closes multiple times before the catalytic cycle is completed. Our model of the enzymatic activity coupled with conformational changes suggests that as long as these rates are substantially higher than the catalytic turnover rate (*k*_cat_), the explicit interconversion rates between the open and closed states do not determine enzyme activity. Instead, the relative population of the two states is what determines activity and inhibition.

A matter of intense debate in enzyme catalysis is whether conformational fluctuations play a role in catalysis (2, 3, 46, 47). While several studies have claimed that enzyme fluctuations play a direct role in the chemical step, theoretical studies have refuted those (48). Our work suggests that the ultrafast domain movements help to tune the equilibrium between states of the enzyme with different activities continuously. Such a mechanism is not unique to AK. For example, we recently discovered that the activity of the AAA+ disaggregation machine ClpB is tightly correlated to the distribution between active and inactive conformational states (49). However, the interconversion between these two states happens on a much faster timescale than any other protein activity (49). Schanda et al. found that the enzymatic activity of the TET2 aminopeptidase is controlled by the conformational equilibrium of a highly dynamic loop in the catalytic chamber (50). Also, the turnover in the HisFH enzyme complex is dictated by the population of the active state, with the conversion between ground and active state taking place on a faster timescale than enzymatic activity (51).

This study goes beyond previous attempts to connect conformational dynamics and chemical steps in enzymes by developing a kinetic model that explicitly takes conformational changes into account. The model builds on the recent in silico observation that fast opening and closing cycles might help to find the correct mutual substrate orientation efficiently and to guarantee the high catalytic activity of the enzyme (23). It is further based on the hypothesis that the order of binding of the two substrates to the enzyme dictates how effectively repeated opening and closing cycles help to reorientate the substrates for the reaction. Strikingly, this model is able to correctly reproduce trends of substrate inhibition among the WT enzyme and several mutants. In conclusion, our study of a common and widely studied enzyme uncovers the mechanistic relationship between very fast conformational variation and catalysis.

## Methods

See *SI Appendix* for a detailed description of the methodology for sample preparation, data collection, and analysis. Briefly, a large library of AK mutants was screened for the strength of substrate inhibition. A total of 86 unique mutants were purified and tested for their activity. The resulting inhibition constants were correlated with the conservation indices of mutated residues. Proteins for smFRET experiments were expressed and labeled with two fluorescent dyes. Single-molecule data were collected on freely diffusing molecules and analyzed using the statistical analysis algorithm H^2^MM, to obtain domain opening and closing rates. These rates served as input for simulation of enzyme kinetic curves using a model that included conformational dynamics.

## Associated Content.

### Supporting information.

Methods section containing a detailed description of the methodology, *SI Appendix*, Figs. S1–S11 and Tables S1–S10, supporting references.

## Supplementary Material

Appendix 01 (PDF)

## Data Availability

All study data are included in the article and/or *SI Appendix*.
