# Supplementary material for: Allosteric communication between ligand binding domains modulates substrate inhibition in adenylate kinase
Source: Proc Natl Acad Sci U S A. 2023 Apr 24;120(18):e2219855120. doi: 10.1073/pnas.2219855120 (PMC10160949; doi:10.1073/pnas.2219855120)
Supplement: Supplementary file 1 — Appendix 01 (PDF) [file pnas.2219855120.sapp.pdf]

Allosteric communication between ligand binding domains modulates substrate inhibition in adenylate kinase

David Scheerer[a], Bharat V. Adkar[b!], Sanchari Bhattacharyya[b], Dorit Levy[a], Marija Iljina[a], Inbal Riven[a], Orly Dym[a], Gilad Haran[a] and Eugene I. Shakhnovich[b]

[a] Department of Chemical and Biological Physics, Weizmann Institute of Science, Rehovot 761001, Israel
[b] Department of Chemistry and Chemical Biology, Harvard University, 12 Oxford St, Cambridge, MA 02138
! equal contribution

Supporting Information

This PDF file includes:

Methods

Supplementary Note 1

Figures S1 to S11

Tables S1 to S10

METHODS

The degenerate library of AK

AK's structure can be divided into three main domains – the ATP-binding LID domain (residues 118-160), the AMP-binding NMP domain (residues 30-67), and the rest, i.e., the CORE domain. The sites for mutations were chosen based on the following criteria: a) the sites should not be contacting any of the active site residues. The active site residues were defined as those residues that show >5 Å^2 change in solvent-accessible surface area with and without the inhibitor, Ap_5A (PDB identifier 1AKE, (1)). b) The sites should be at least 8 Å away from Ap_5A. c) The side-chain of the WT residue should not be involved in any salt bridges or hydrogen bonds with other protein residues. 71 residues satisfied all these criteria, of which 55 were in the CORE, 10 in the LID, and

6 in the NMP domain. A degenerate codon "DHK" [D = A or T or G][H = A or T or C][K = T or G] was used to generate 13 different amino acid mutations at each of these selected 71 sites. The DHK library at each site represents all amino acid types (AILMV+DE+FY+K+NST + 1 stop codon) and hence offers a convenient alternative to "NNK" – the fully degenerate codon that codes for all 20 amino acid diversity. The mutational library at each position was generated separately by amplifying the whole plasmid (pET28a(+) with AK gene cloned between *Nde*I and *Xho*I) with the mutagenic primers. The PCR products were pooled together and transformed in *E. coli* DH5α as a single library. A single plasmid prep was done and transformed in the expression host *E. coli* BL21(DE3). A total of 1248 colonies were picked up for further assessment.

Activity assay from cell lysate

All 1248 library candidates were grown overnight in LB in thirteen 96-well plates. This culture was diluted 1:100 in 500 µl of LB in 2 ml deep-well plates. The cultures were induced with 1 mM IPTG at OD600~0.4 (~3h) and further grown overnight before they were pelleted. The cell lysis was done using BugBuster/Benzonase mix per the manufacturer's instructions. The cell lysate was diluted 1:4000 in 10 mM potassium phosphate buffer, pH 7.2, and used for enzyme activity as described previously (2). A quick assessment of the inhibition status was possible from the ratio of v_0 at 100 and 500 µM of AMP (ATP was fixed at 1000 µM) as shown in Fig. S1b.

High-throughput protein purification

A total of 192 candidate proteins were purified – 64 each from the right, left and the middle of the distribution of v_0^{100}/v_0^{500}. The proteins were expressed in 500 µl LB and lysed as described earlier. The his-tagged protein from the lysate was purified using HisPur™ Ni-NTA spin plate (ThermoFisher, cat # 88230). Imidazole was removed from the eluted protein solution using Zeba™ Spin desalting plate (7K MWCO) (ThermoFisher, cat # 89808), and the proteins were desalted into a 10 mM potassium phosphate buffer, pH 7.2. A first pass at Sanger's sequencing confirmed 86 unique mutants with reliable chromatograms. Enzymatic activity assays were carried out as described previously (2). Values for the Michaelis constant (K_M) and an effective inhibition constant (K_I) were obtained by a fit to

$$v_0 = \frac{v_{max} c}{K_M + c\left(1 + \frac{c}{K_I}\right)} \quad (1)$$

where c is the concentration of AMP and v_{max} the maximum turnover.

Subsequently, we compared the strength of inhibition (i.e. the size of K_I) to the conservation index of the respective mutated amino acid. High conservation refers to a high frequency of occurrence in a multiple-sequence alignment (3, 4)). Using a Student t-test, we compared locations of mutations that led to a loss of inhibition (K_I >=1500) to locations that led to an increase of inhibition (K_I <=500). Positions where both inhibited and uninhibited mutations were observed (magenta in Table S1) were not considered in this analysis.

Expression and labeling of proteins for fluorescence experiments

For the smFRET experiments, we chose proteins from the group above that demonstrated a similar or slightly higher stability than the WT (2, 5). A pET15b expression vector containing the E. coli AK C77S gene with a six-residue histidine tag at the N terminus was used to express the protein for fluorescence experiments. Alanine-to-cysteine and valine-to-cysteine substitutions were introduced at positions 73 and 142 of the protein, respectively, by site-directed mutagenesis. The sequence of AK C77S/V142C/A73C was verified by DNA sequencing. The mutant was overexpressed by transformation into E. coli BL21 competent cells. The cells were lysed using high-energy sonication or a French cell press. The pellet was then separated from the protein-containing supernatant using high-speed centrifugation. The supernatant was separated on a Ni Sepharose column (GE Healthcare HisTrap HP). Fractions containing AK were pooled and run on a second gel filtration column (HiLoad 16/60 Superdex 75 prep grade; GE Healthcare) and eluted as a single peak. Labeling reactions were performed by first incubating protein samples with Alexa 594 maleimide (Invitrogen) at a molar ratio of 75%, separating labeled from unlabeled protein on a mono-Q 5/50 GL column (GE Healthcare), and later-on labeling with an excess of Alexa 488 maleimide (Invitrogen).

Enzymatic activity measurements of labeled protein

The enzymatic activity for the purified and the double-labeled protein variants was conducted at pH 8.0, mimicking the conditions of the single-molecule experiments. The velocity was monitored for the forward reaction (MgATP+AMP → ADP +MgADP) by the oxidation of NADH at 340 nm in coupling with pyruvate kinase and lactate dehydrogenase. The final assay mixture was: 4 nM AK, 50 mM Tris-HCl (pH 8.0), 100 mM KCl, 4 mM phosphoenolpyruvate, 5.0 mM $MgCl_2$,

0.2 mM NADH, 10 units/mL pyruvate kinase, 15 units/mL lactate dehydrogenase, 0.25 mg/mL bovine serum albumin, 1 mM ATP and varying concentrations of AMP. The initial velocity was obtained by linearly fitting the NADH signal as a function of time. For the forward direction, the initial velocity of the reaction v_0 was fitted to the model for two kinetically distinct pathways (6, 7):

$$v_0 = \frac{ic^2 + jc}{1 + lc^2 + mc} \tag{2}$$

where c is the concentration of AMP i, j, l and m are functions of the ATP concentration (6) and the various steps shown in Fig. 5a.

Structure determination and refinement of AK L107I in complex with Ap_5A

Crystals of AK L107I in complex with Ap_5A were obtained using the sitting-drop vapor-diffusion method with a Mosquito robot (TTP LabTech). The crystals were grown from 7.5% PEG 3350, 7.5% PEG 4000, 7.5% PEG 2000, 7.5% PEG 5000 monomethyl ether, 0.07M ammonium nitrate, 2.5% ethylene glycol and 0.05M MES pH=7. The crystals formed in the space group $P22_12_1$, with six copies per asymmetric unit. A complete dataset to 2.05Å resolution was collected at 100 K from a single crystal on an in-house liquid-metal-jet (LMJ) X-ray diffractometer.

Diffraction images were indexed and integrated using the CrysAlis Pro software from Rigaku, and the integrated reflections were scaled using the SCALA program (8). Structure factor amplitudes were calculated using TRUNCATE (9) from the CCP4 program suite. The structure was solved by molecular replacement with the program PHASER (10), using the structure of AK from *E. Coli* (PDB code 4JZK).

All steps of atomic refinement of both structures were carried out with the CCP4/REFMAC5 program (11) and by Phenix refine (12). The models were built into $2mF_{obs}$ - DF_{calc}, and mF_{obs} - DF_{calc} *maps* by using the COOT program (13). Details of the refinement statistics of the AK L107I mutant in complex with Ap_5A structure are provided in Table S10. The coordinates were deposited in the RCSB Protein Data Bank (PDB:8BQF).

smFRET data acquisition

Single-molecule data was acquired on freely diffusing molecules using a Microtime 200 system (PicoQuant). Flow cells were prepared as described previously (14) and filled with a mixture of 15 pM labeled enzyme, 50 mM Tris-HCl (pH 8.0), 100 mM KCl, 5 mM $MgCl_2$, 0.01% Tween (Thermo Fisher), and substrates (ATP, ADP, AMP; Sigma). Importantly, we verified that our ATP solutions did not contain any ADP using ^{31}P-NMR spectroscopy. Substrate concentrations used for experiments under turnover conditions are given in Table S5. The appropriate ADP concentration to guarantee equilibrium (zero flux) was calculated using the following rate equation:

$$v \propto \frac{k_1[M][T]}{K_{D,T}K_{D,M}} - \frac{k_{-1}[D1][D2]}{K_{D,D1}K_{D,D2}} \tag{3}$$

where k_1/ k_{-1} are the forward and backward rate constants for the reaction and [M] and [T] are the AMP and ATP concentrations. $K_{D,S}$ is defined as the dissociation constant of the respective substrate (T,M,D1,D2) and were taken from Sheng *et al.* (15). [D_1] and [D_2] are the concentrations of ATP bound to the LID and NMP domain, respectively. [D_2] is calculated using

$$[D_2] = \frac{\frac{1}{K_{Mg}} + [D] + [Mg] - \sqrt{\left(\frac{1}{K_{Mg}} + [D] + [Mg]\right)^2 - 4 \cdot [D] \cdot [Mg]}}{2} \tag{4}$$

where [Mg] and K_{Mg} (15, 16) are the concentration and dissociation constant for magnesium, respectively. FRET efficiency histograms from the first and last 1h of each measurement were shown to overlap, validating that indeed the substrate concentration did not change during the measurement.

Measurements were conducted in the pulse-interleaved excitation mode, using a sequence of one pulse for acceptor excitation (594 nm, power 10 μW) and three pulses for donor excitation (488 nm, power 50 μW) at 40 MHz. The laser beams were focused 10 μm deep into the sample solution. Molecules diffusing through the beam emitted short bursts of photons that were divided into two channels according to their wavelengths, using a dichroic mirror (zt594rdc; Chroma) and filtered by band-pass filters (HC520/35 (Semrock) for the donor channel and ET 674/75m (Chroma) for the acceptor channel). Arrival times of these photons were registered by two single-photon

avalanche photodiodes (SPCM-AQRH-14-TR, Excelitas) coupled to a time-correlated single-photon counting module (PicoHarp 400, PicoQuant).

Photon burst selection

Fluorescent bursts in the single-molecule data were detected using methods developed in the lab (14, 17, 18). A cut-off time of 5 μs between individual photons was determined from the histogram of the time lags and used to find the effective start and end points of each burst. Only fluorescent bursts with a total of 50 photons or more were selected for further analysis, resulting in an average photon flux of around 400 photons per millisecond. The raw FRET efficiency of each burst was calculated based on the photons detected in both channels following donor excitation only. The raw stoichiometry was obtained from the detected photons in both channels after both excitations, as described elsewhere (19, 20). A 2D histogram of raw stoichiometry versus raw FRET efficiency was generated, from which we extracted the amount of emitted donor photons leaking into the acceptor channel and the level of direct excitation of the acceptor dye by the 485 nm laser. The photon stream in both channels was corrected for the leakage of photons from the donor to acceptor, using the apparent FRET efficiency of donor-only molecules (19, 21). The correction factor for direct excitation of the acceptor dye was determined from the acceptor-only population (19, 21). The FRET efficiency for each burst was calculated from the corrected number of photons arriving from the acceptor channel divided by the total number of photons. To obtain the final FRET histogram without the donor-only and acceptor-only populations, we selected only photon bursts with a stoichiometry corresponding to molecules with both donor and acceptor dyes.

Analysis of protein dynamics with H^2MM

To extract the dynamics hidden in the photon bursts, we used the H^2MM algorithm (17). Only double-labeled molecules and photons arising from donor excitation were taken for this analysis. For each protein variant, the FRET efficiencies of the open and closed state were optimized globally to give the best fit across varying substrate concentrations. This procedure was employed since the structures of the two states themselves were considered to be unaltered by substrate binding, in contrast to the distribution between the states. On the other hand, the initial populations of the states and the interconversion rates were optimized for each measurement separately. Similar FRET efficiency values were obtained for all mutants, 0.37±0.02 / 0.72± 0.02 for the WT

and L107I, 0.38±0.01 / 0.68±0.02 for L82V and 0.40±0.01 / 0.70±0.02 for F86W for the open and closed state, respectively. This reflects the fact that the mutations do not significantly alter the overall protein structure. In the case of L107I, this could be additionally verified by the excellent agreement of the crystal structure of the closed state reported in this work with that of the WT (1).

For each mutant, at least two different data sets (independently prepared samples) were analyzed for all different substrate concentrations. The resulting parameters were validated with different tests: A recoloring analysis (Fig. S5), a visualization of the impact of the interconversion rate on the FRET efficiency histogram (Fig. S6), a dwell-time analysis (Fig. S7, Table S3) and fluorescence correlation spectroscopy experiments (Fig. S8, Table S4). Details are given in the paragraphs below. Representative single-molecule trajectories are shown in Fig. S4. These include the assignment of the most likely state-sequence using the Viterbi algorithm on a photon-by-photon basis (17).

Our kinetic model does not take into account dye blinking, as blinking events are largely filtered out in our rigorous burst selection. Indeed, less than 0.1% of our selected trajectories show a maximum interphoton times of more than 100 μs after acceptor excitation. Blinking of the acceptor dye would manifest as gaps in this photon stream. As a test, we have also carried out H^2MM analysis on data sets where bursts with a potential blinked state ($\Delta t>100$ μs after acceptor excitation) had been filtered out, and saw minimal differences compared to the complete data sets. For a visualization of the photon streams after both donor and acceptor excitation, see Fig. S4.

Recoloring analysis

We performed a recoloring analysis to verify the parameters obtained from the H^2MM analysis (14, 22). In this method, the arrival times of photons in each data set are retained, but their “colors” (i.e. whether they belong to the donor or acceptor) are erased. A stochastic simulation based on the H^2MM parameters is then used to reassign the photons to the two experimental channels, and FRET efficiency histograms are reconstructed. A good match between the original histograms and the recolored ones indicates a successful analysis.

Dwell-time analysis

The dwell-time analysis yields the distributions of times that the protein spends in each state (in this case, the open and closed state). Here we computed these he distributions using a likelihood-weighted segmentation algorithm developed in-house (17, 23). In this analysis, for each burst every possible sequence of states contributes to each dwell time a fraction of a count, equal to the likelihood of the sequence. In contrast with the more common dwell-time analysis based on the Viterbi algorithm, here all possible state sequences are taken into account, not only the most likely one. A good agreement of rates obtained directly from the H^2MM analysis and those obtained from the dwell-time analysis was considered as a validation of the analysis (Table S3).

Fluorescence correlation spectroscopy

FCS experiments were performed on freely diffusing molecules using the Microtime 200 microscope (PicoQuant) at a power of 20 μW (488 nm), at a concentration of 1 nM of the double-labeled WT protein and varying concentrations of ATP. For the cross-correlation between donor and acceptor photons, identical optical elements to those mentioned in the chapter "smFRET data acquisition" were used. Auto-correlation between donor photons was obtained by first filtering the light using a band-pass filter (HC520/35 (Semrock) and then splitting it between two detectors (to reduce the effect of afterpulsing). Correlation functions were calculated using the SymPhoTime 64 software (PicoQuant). To isolate the conformational dynamics from the diffusion contribution to the correlation functions, for each sample we calculated the ratio between the cross-correlation of donor and acceptor (CC_{DA}) and the auto-correlation of donor photons (AC_{DD}), based on the procedure of Torres and Levitus (24):

$$\frac{CC_{DA}}{AC_{DD}} = \frac{\frac{1}{N_{DA}} * G_{diff}(t) * G_{DA}(t)}{\frac{1}{N_{DD}} * G_{diff}(t) * G_{DD}(t) * G_{trip}(t)} = \frac{1}{N} * \frac{G_{DA}(t)}{G_{DD}(t) * G_{trip}(t)} \quad (5)$$

This operation cancels out the diffusion term $G_{diff}(t)$ in CC_{DA} and AC_{DD}. N_{DA},N_{DD} describe the number of molecules containing both labels and the donor label, respectively. These numbers differ due to the presence of donor-only labeled molecules and photobleaching (25). We combine both terms into an effective amplitude N. The remaining terms attribute for conformational

($G_{DA}(t)$, $G_{DD}(t)$) as well as triplet dynamics ($G_{trip}(t)$). Once the diffusion term is cancelled, we use the following equation for fitting:

$$\frac{CC_{DA}}{AC_{DD}} = \frac{1}{N} * \frac{\left(1 - K * exp\left(\frac{-t}{\tau_c}\right)\right)}{\left(1 + \frac{p}{1-p} * exp\left(\frac{-t}{T}\right)\right)} \tag{6}$$

τ_c is the time constant for conformational dynamics. The pre-exponential factor K depends on kinetic rates and the relative visibility of each state (24). T is the triplet relaxation time and p the fraction of dye molecules in the triplet state. T and p are independent of substrate concentration and were optimized globally.

Differential scanning fluorimetry

Temperature melts of AK variants were carried out using a BioRad CFX384 real time PCR machine with the SYPRO orange dye. The final concentration of protein used was 4 μM, and the solution contained SYPRO orange at 5×, 5 mM of $MgCl_2$, and varying concentration of ATP (0-20 mM) or AMP (0-40 mM). The total volume was adjusted to 10 μl with 10 mM potassium phosphate buffer, pH 7.2. The melting temperature (Tm) was estimated as the temperature at which the melt-curve derivative peaks. This method yields a proxy for binding as it essentially determines the stabilization of a protein by its ligand. The effect of mutation on ATP or AMP can be qualitatively determined from curves of Tm as a function of the ligand concentrations.

Simulation of the enzymatic activity

Activity curves were simulated with the program KinTek Kinetic Explorer (KinTek, Snow Shoe, PA), with experimentally derived protein dynamics parameters and substrate affinities as input. Opening and closing rates were obtained by fits to the apparent rates observed in the smFRET experiments, as described in Supplementary Note 1. The obtained parameters are given in Table S8. The rate of productive binding events (k_1) was derived from the enzymatic activity data, using the ratio k_{cat}/K_M (often referred to as the specificity constant). With the Michaelis constant K_M given as

$$K_M = \frac{k_{-1} + k_{cat}}{k_1} \tag{7}$$

and k_{-1} as the rates for substrate release, k_{cat}/K_M can be written as

$$\frac{k_{cat}}{K_M} = \frac{k_1}{k_{-1} + k_{cat}} * k_{cat} = \frac{k_{cat}}{k_{-1} + k_{cat}} * k_1 \qquad (8)$$

and approaches k_1 when the rate of unproductive product release k_{-1} is minimal. The resulting parameters are given in Table S9.

Supplementary Note 1. Substrate dependency of the closed state occupancy. The occupancy of the closed state in AK is dependent on the substrate concentration (Figure 3). In particular, the binding of ATP stabilizes the closed conformation. To account for this behavior, we assume that each substrate-bound species in Figure 5a has a specific distribution between the open and closed conformation.

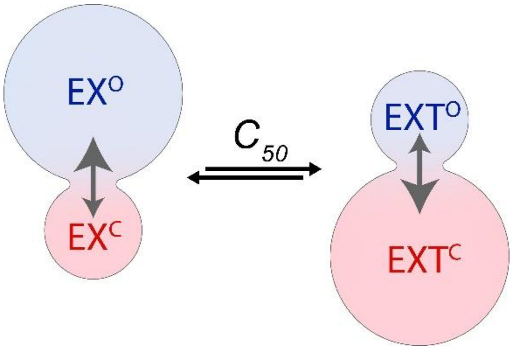

In this scheme, EX denotes the enzyme in its apo (EX=E) or AMP bound (EX=EM) form, and EXT is the enzyme already bound with ATP. The uppercase O/C indicates the protein conformation, open or closed, respectively. In the open state, the enzyme can bind or release ATP. The ratio between the different forms is given by the substrate concentration and $C_{50,ATP}$,i.e. the concentration which triggers half of the maximal conformational change. Each enzyme form contributes to the apparent closed state occupancy Occ_{app} according to its population.

$$Occ_{app} = EX \cdot Occ_{EX} + EXT \cdot Occ_{EXT} = \frac{C_{50,ATP} \cdot Occ_{EX} + [S] \cdot Occ_{EXT}}{C_{50,ATP} + [S]} \qquad (9)$$

with *[S]* as the substrate concentration. *[S]* hereby refers to ATP, when it is the sole substrate, or ATP+ADP when all three substrates are present. Parameters obtained from this model are given in Table S6.

For the addition of AMP to the ATP bound form (ET), we found that minor concentrations (<1 mM AMP) did not induce changes. Therefore, our single-molecule experiments cannot distinguish between the ET state and the non-inhibited ETM state. In contrast, inhibitory concentrations of AMP above 1 mM trigger an increase in the apparent closing rate. We attribute the faster closing to the inhibited $\mathrm{ETM}_{\mathrm{inh}}^{\mathrm{C}}$ state. Similar to Eq. (9), in this scheme each enzyme form contributes to the apparent closing rate $k_{c,app}$ according to the population of the inhibited E_{inh} and non-inhibited E_{non} states (ET+ETM).

$$k_{c,app} = E_{non} \cdot k_{c,non} + E_{inh} \cdot k_{c,inh} = \frac{C_{50,AMP} \cdot k_{c,non} + [M] \cdot k_{c,inh}}{C_{50,AMP} + [M]} \tag{10}$$

with *[M]* as the AMP concentration. Parameters obtained from this model are given in Table S7.

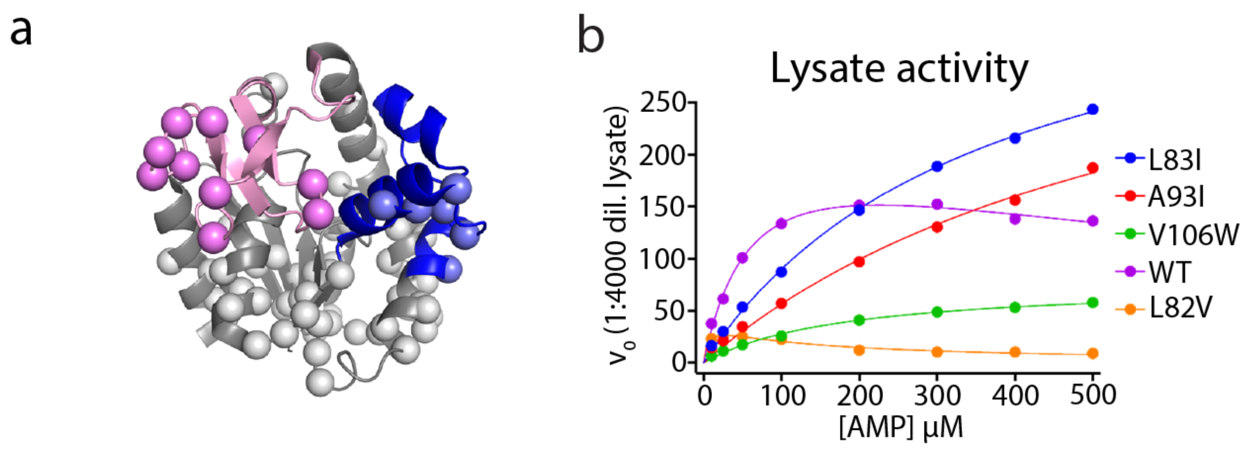

Figure S1: Construction and analysis of the AK mutant library. a) The domains are colored as Core in gray, LID in pink, and NMP in blue. Spheres represent positions chosen for library generation. 71 residues were selected that were at least 8 Å away from the inhibitor AP_5A and whose sidechains were not involved in any hydrogen bonding with rest of the protein. 55 of these positions are present in the Core domain, 10 in the LID domain and 6 in the NMP domain. b) In-lysate activity measurements of known mutants of AK confirms that such measurements can reproduce the inhibition profile of the corresponding purified proteins.

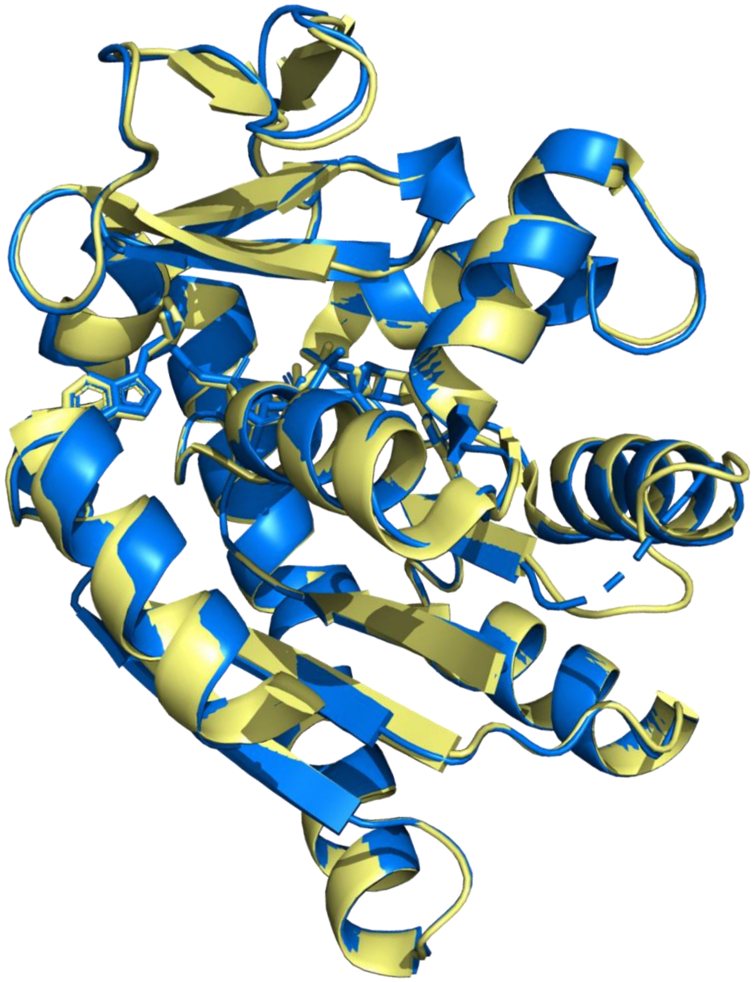

Figure S2: Overlay of the crystal structure of the WT in yellow (PDB:1AKE (1)) with the L107I mutant (blue). Both structures are in complex with the ligand Ap_5A. Shown is the respective first unit of the asymmetric unit.

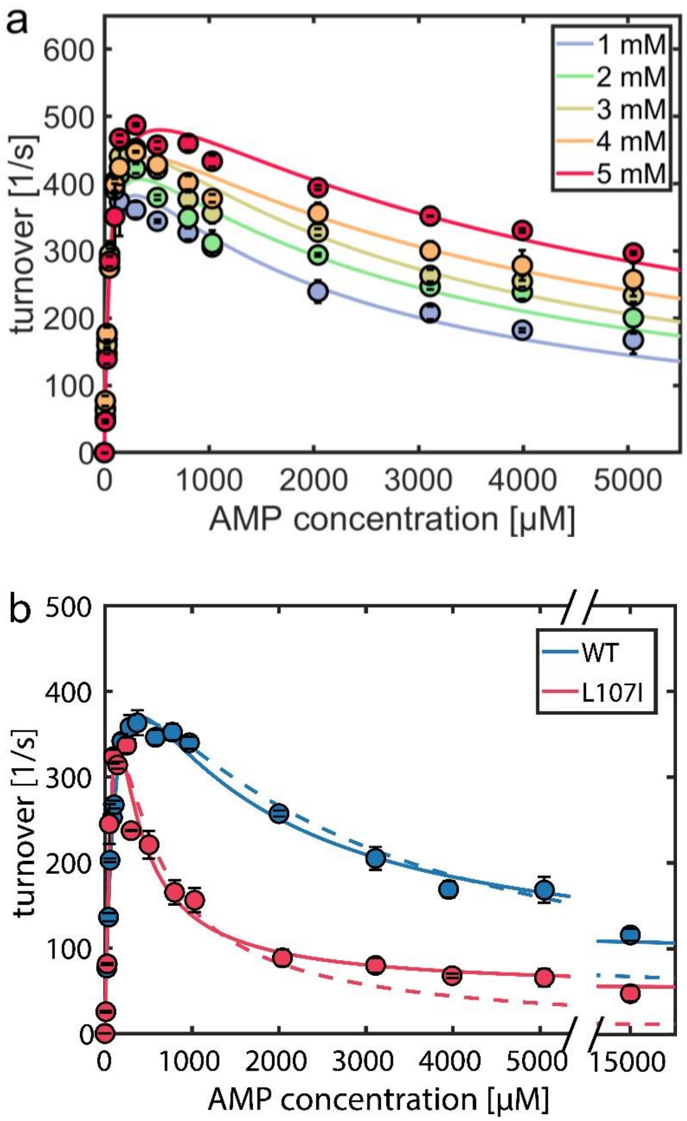

Figure S3: Impact of ATP and AMP on inhibition. (a) ATP lifts substrate inhibition by AMP in a dose-dependent manner for the WT. Kinetic parameters based on an inhibition mechanism involving a dead-end complex are given in Table S2. (b) Enzymatic activity for WT (blue) and L107I (red) as a function of AMP concentration. Solid curves indicate fits for a model with two alternating pathways as described (6). For comparison, fits to an inhibition mechanism with dead-end complexes are shown as dashed lines. The enzyme shows residual activity at high AMP levels, in agreement with previous studies (5, 26, 27), suggesting that the loss in activity cannot be attributed to the formation of a dead-end complex (e.g. AMP bound to the ATP site) but to a kinetically impaired pathway. At low AMP levels, only a slight deviation between the two models is seen.

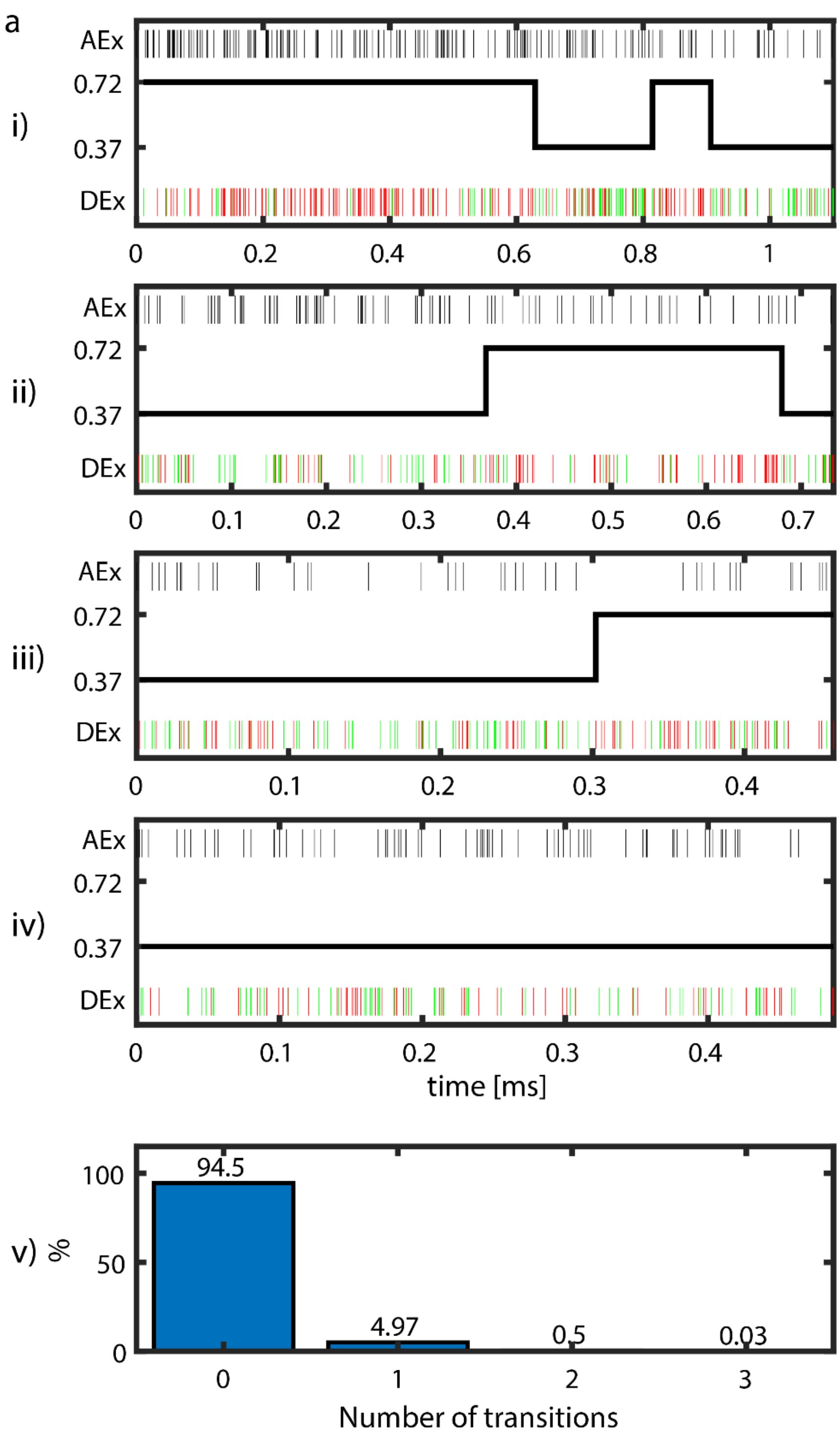
a
i)
ii)
iii)
iv)
v)
AEx
DEx
0.72
0.37
0
0.2
0.4
0.6
0.8
1
0.1
0.3
0.5
0.7
time [ms]
94.5
4.97
0.5
0.03
100
50
%
Number of transitions
2
3

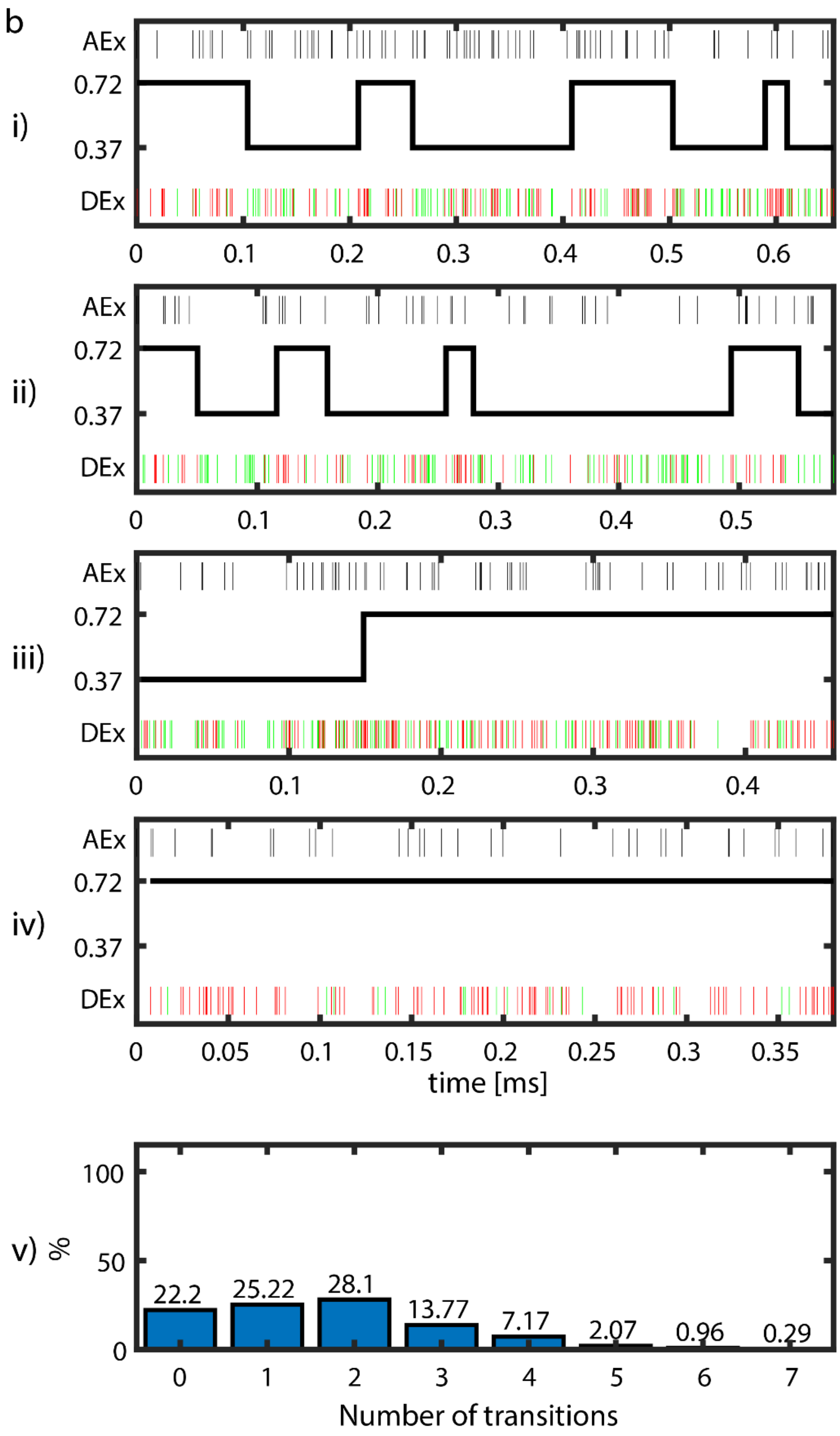

Figure S4: Representative photon-by-photon trajectories and Viterbi assignment. Representative single-molecule trajectories are shown for the WT protein under a) the apo condition and b) 1mM ATP. For i-iv), the top panel shows the arrival time of photons after the

acceptor pulse. The bottom panel shows the arrival time of photons after the donor pulse, in green for donor photons and red for acceptor photons. The black solid line depicts the most likely state sequence according to a Viterbi assignment (17). v) depicts the distribution of the number of transitions identified by the Viterbi algorithm in each data set. Under the apo condition (a), only a few trajectories show more than one transition (i+ii). Most trajectories show either one (iii) or no transition (iv). In the presence of ATP (b), domain opening and closing are faster (Table S3), increasing the probability to observe a transition within a single trajectory.

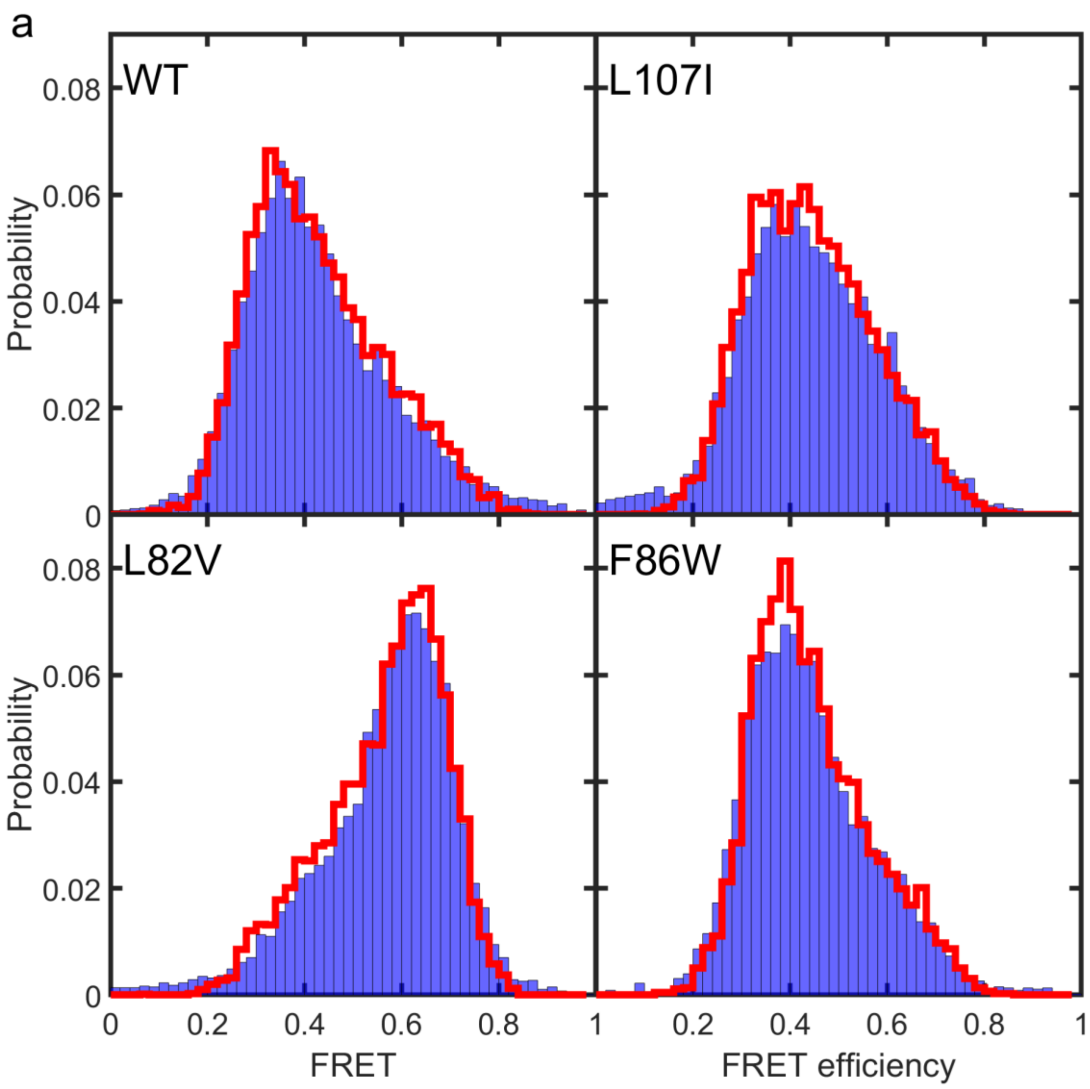
a
WT
L107I
L82V
F86W
Probability
0.08
0.06
0.04
0.02
0
0.2
0.4
0.6
0.8
1
FRET
FRET efficiency

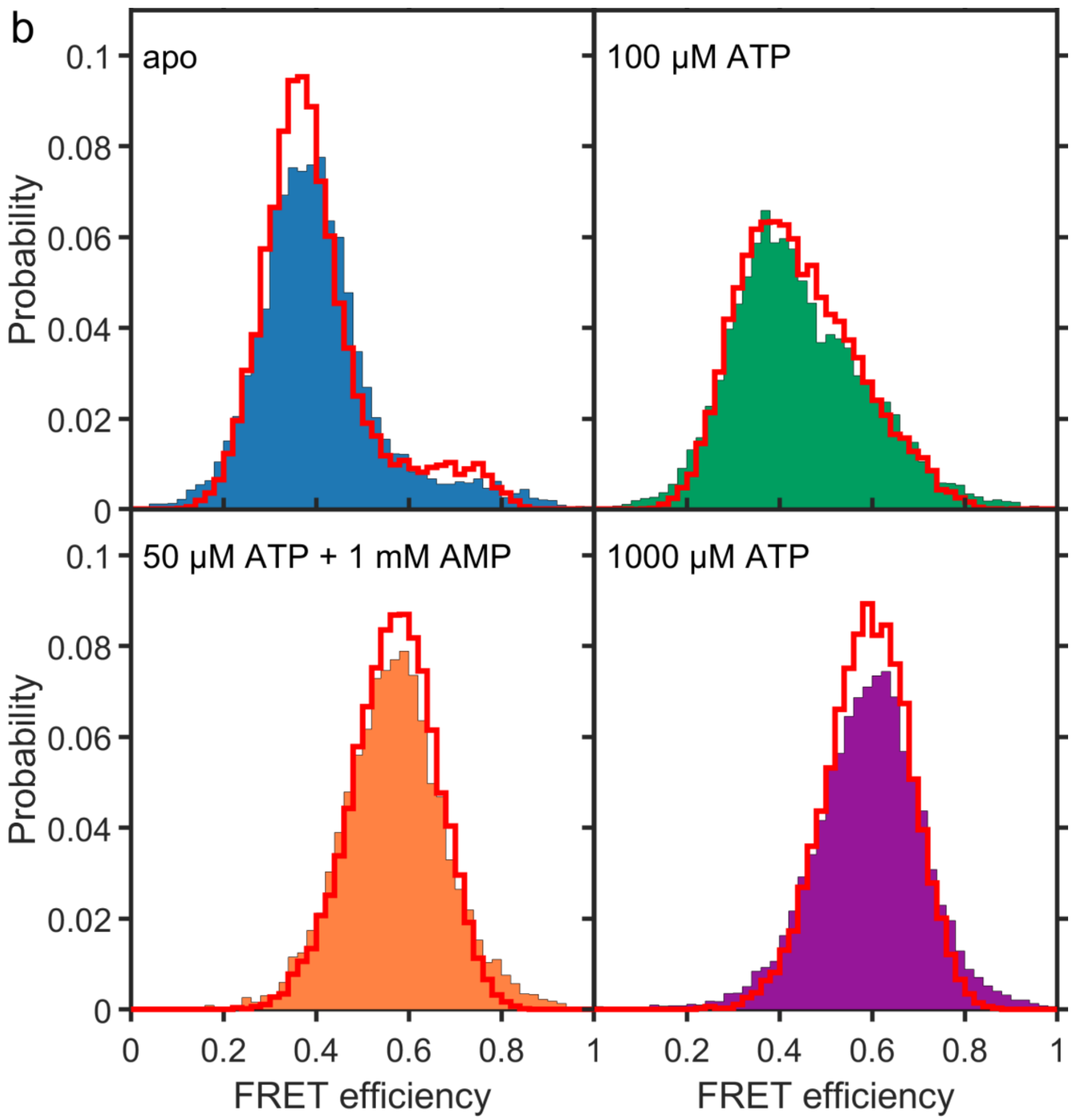

Figure S5: Validating H^{2}MM models using recoloring. a) Representative experimental histograms for the different protein variants are shown in blue at an ATP concentration of 100 µM. The recolored histograms are depicted as solid red lines and show very good agreement between simulation and experiment. b) The model also attributes well to the effect of substrate binding. Shown are recolored histograms as red lines for the WT at the substrate concentrations depicted in Fig. 2a, in matching colors: blue for the apo protein, green for 100 µM ATP, orange for 50 µM ATP with 1 mM AMP (orange) and purple for 1000 µM ATP.

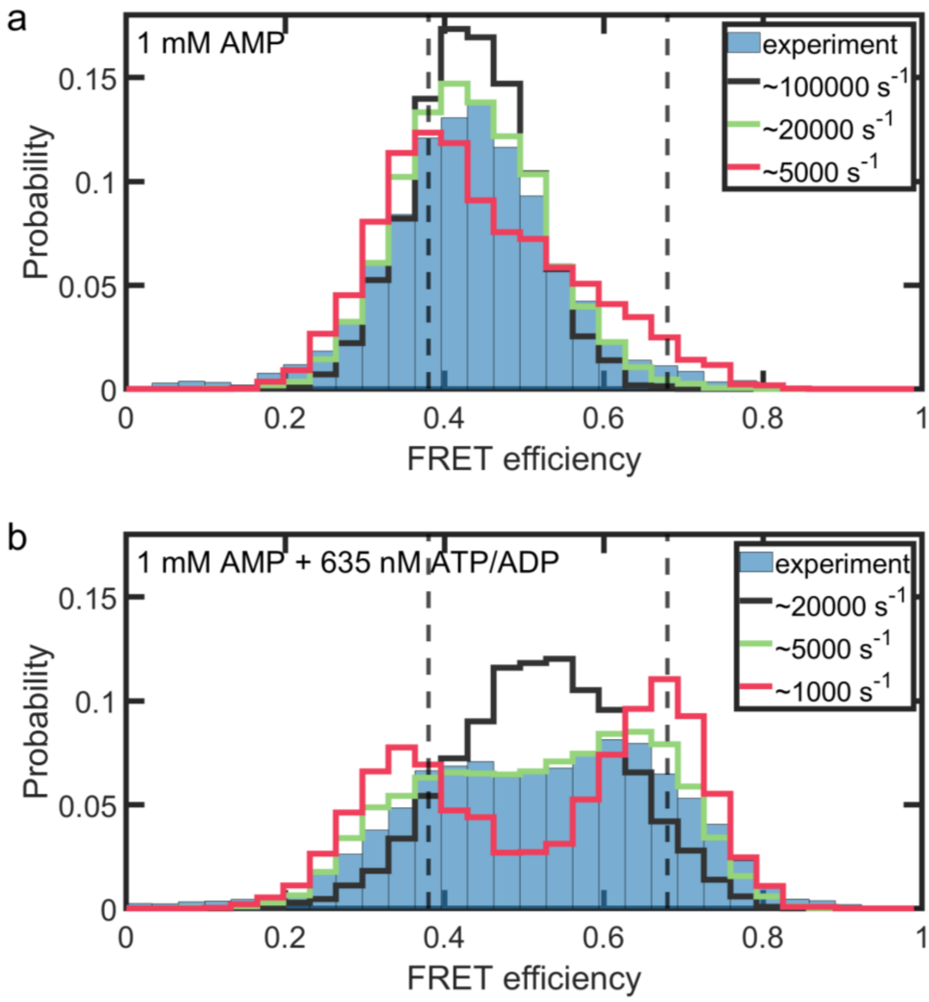

Figure S6. Visualizing the effect of interconversion rates on the FRET histogram. The experimental FRET histogram of L82V (blue) is plotted together with recolored histograms, reflecting the distribution of open/closed states from H^2MM analysis but with altered interconversion rates between the states. The dashed lines indicate the most likely positions of the open (0.38) and closed state (0.68) for L82V obtained from the analysis. The green curve depicts the histogram based on the rates from the H^2MM analysis, the black curve is for 4 times faster conversion rates, and the red curve for 4 times slower rates. (a) The protein shows a fast interconversion in the presence of AMP alone, with the conformational equilibrium in favor of the open conformation. (b) Adding a minor amount of ATP/ADP (combined concentration 625 nM) shifts the population towards the closed state (compare to Fig. 3c). Due to the slower interconversion, the histogram splits up into two peaks, as the average dwell time of the states

exceeds the average burst length. In both a+b), the rates derived by H^2MM (green) give the best fit to the experimental data.

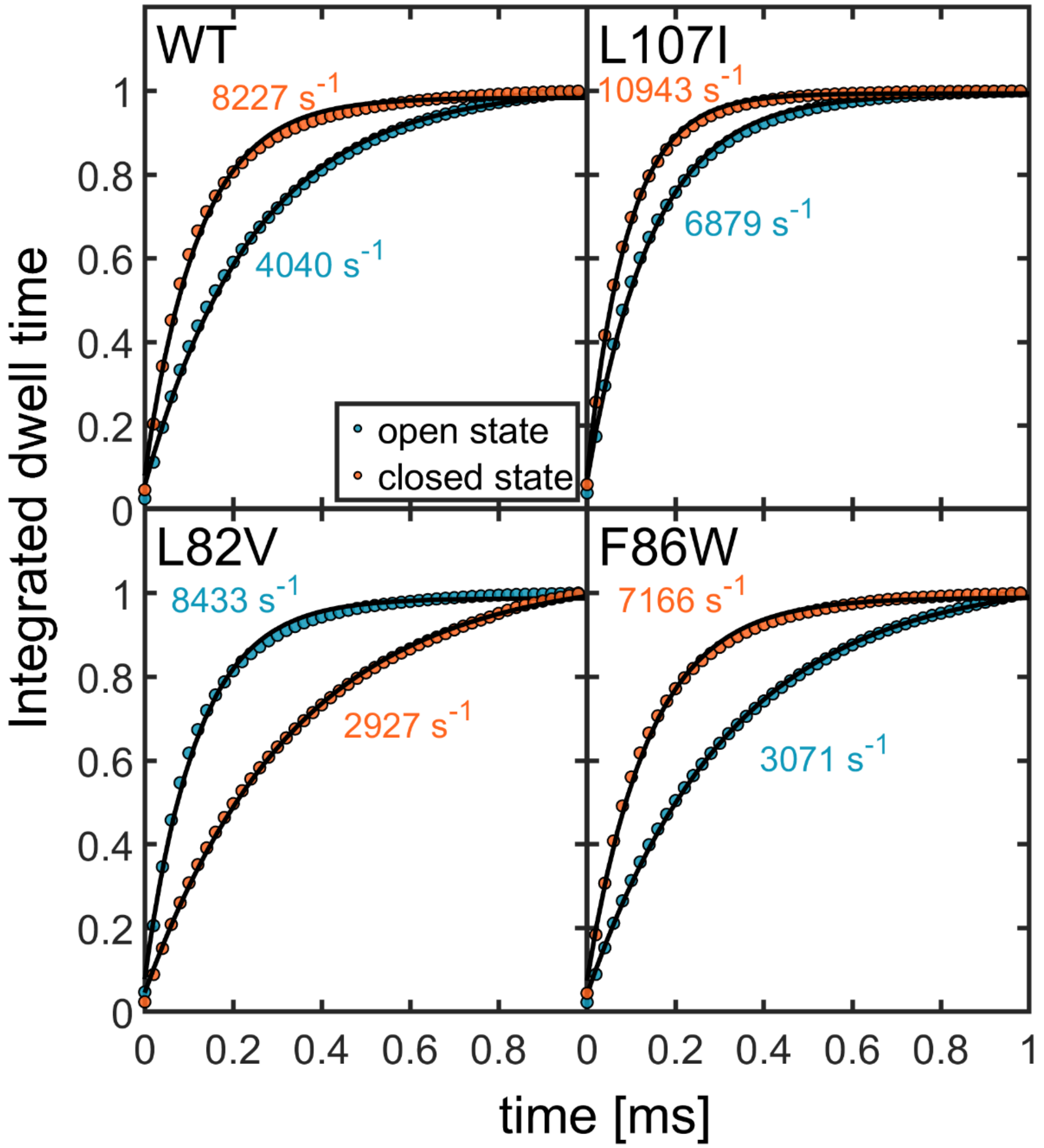

Figure S7. Dwell times analysis. Integrated dwell-time distributions are shown for the open state (cyan) and the closed state (orange) for the four different protein mutants as in Fig. S6a (ATP concentration 100 µM). Black lines are fits to single-exponential functions. Both closing and opening rates extracted from the dwell time distributions compare very favorably to the rates obtained by H^2MM analysis (Table S3).

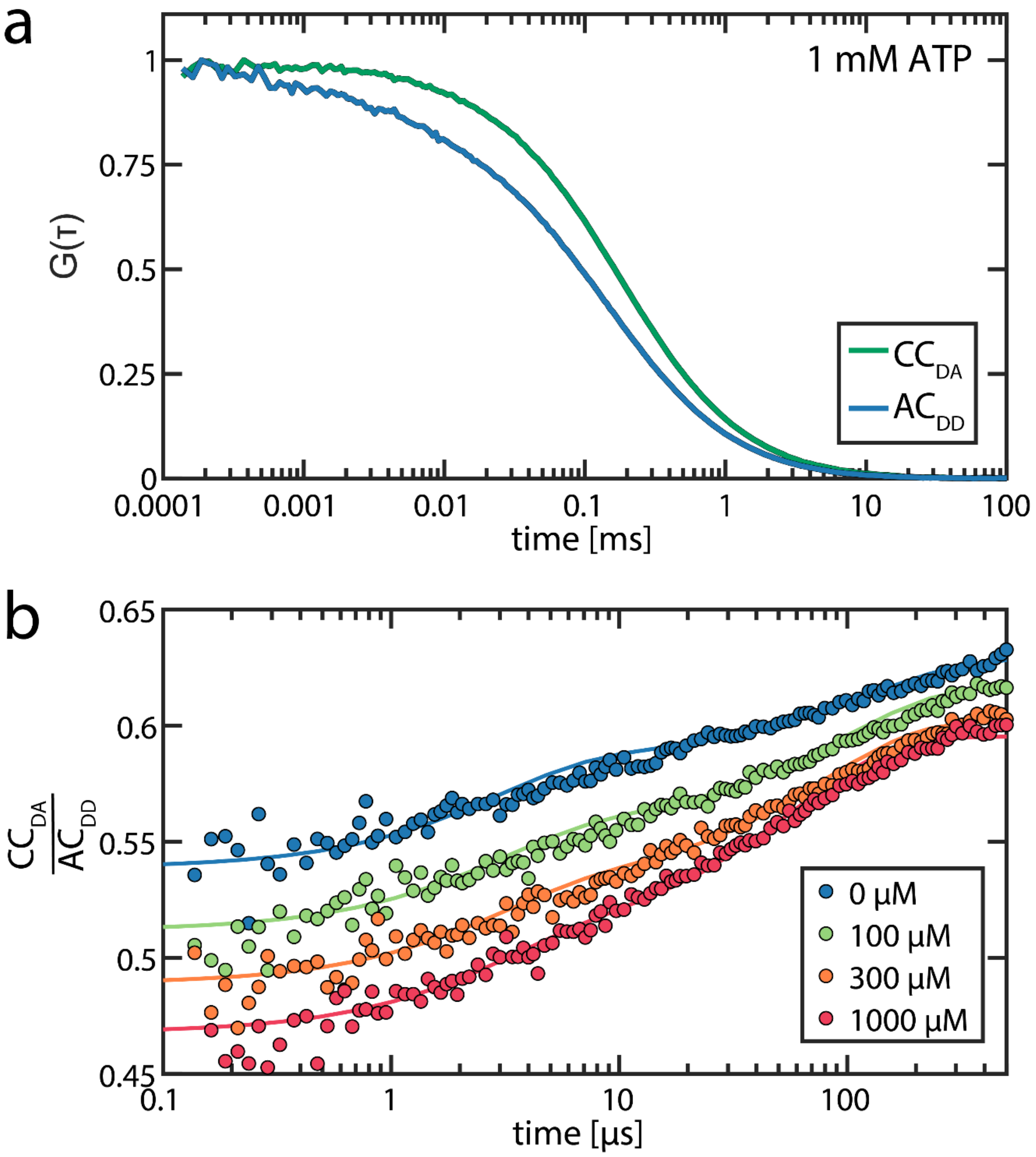

Figure S8. Fluorescence correlation spectroscopy. As an independent control for the presence of fast conformational dynamics in AK, we performed fluorescence correlation spectroscopy on the double-labeled WT protein. (a) Green- donor and acceptor cross-correlation (CC_{DA}) at an ATP concentration of 1 mM, blue- donor auto-correlation (AC_{DD}). Curves were normalized for a better comparison and show the loss of correlation due to the diffusion of molecules. In AC_{DD}, correlation is lost faster due to the presence of conformational dynamics on the microsecond timescale (and triplet kinetics). b) To isolate the conformational dynamics from the diffusion part, the ratio

CC_{DA}/AC_{DD} is calculated (28). Shown are measurements at ATP concentrations of 0 µM (blue), 100 µM (green), 300 µM (orange) and 1000 µM (red). The curves were fit to equation 6, parameters are given in Table S4. With increasing ATP concentration, the time constant for conformational dynamics τ_c decreases as the conformational dynamics are getting faster, in agreement with our H^2MM analysis. Simultaneously, the amplitude K increases.

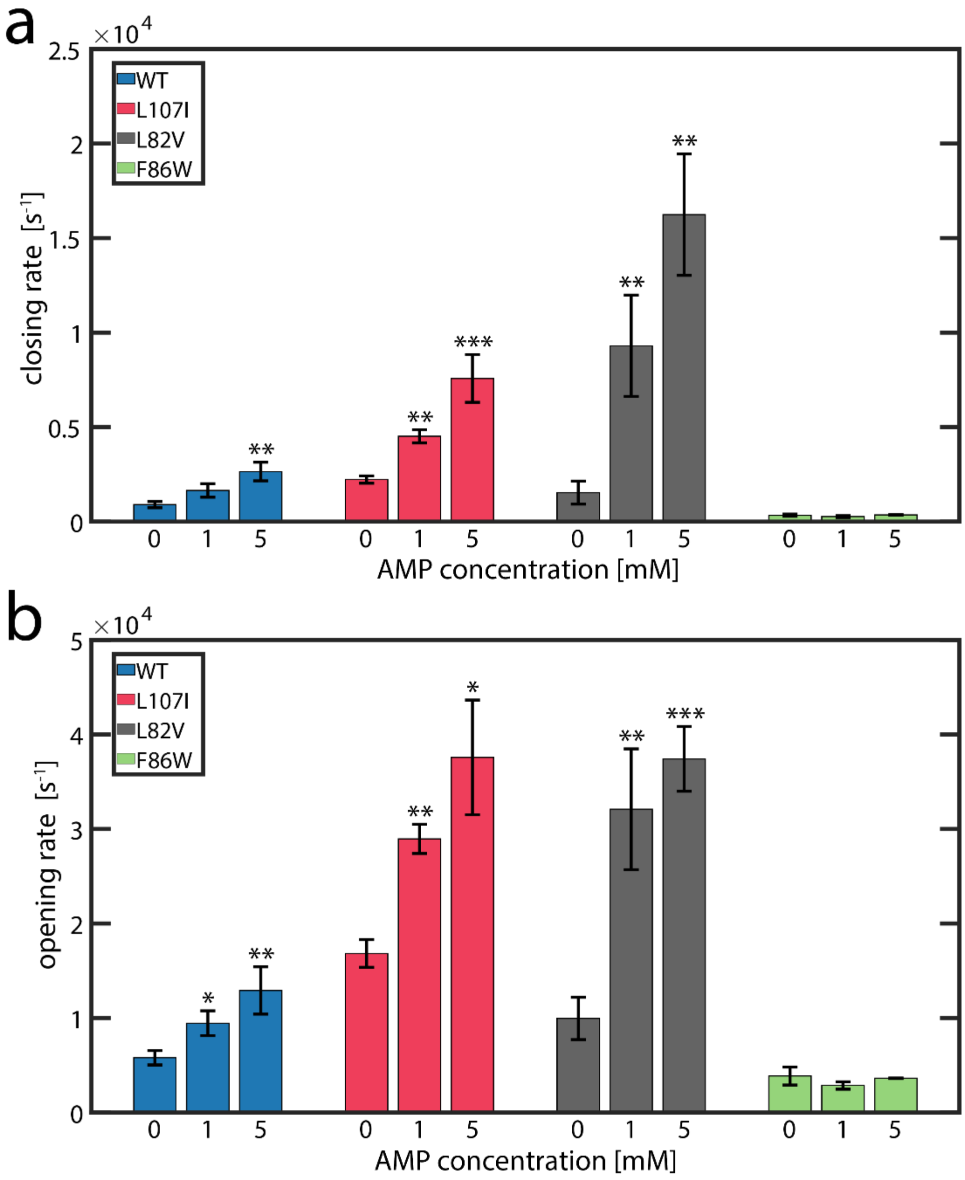

Figure S9: Inhibited protein variants show faster protein dynamics in presence of AMP. Closing (a) and opening (b) rates are altered for all mutants, except for the non-inhibited F86W. Asterisks indicate the significance of the deviation of parameters from the apoprotein (***: p<0.01, **: p<0.05, *: p<0.1, no index: p>0.1, t-test). Error bars are given as the standard error of the mean of 3 independently prepared samples.

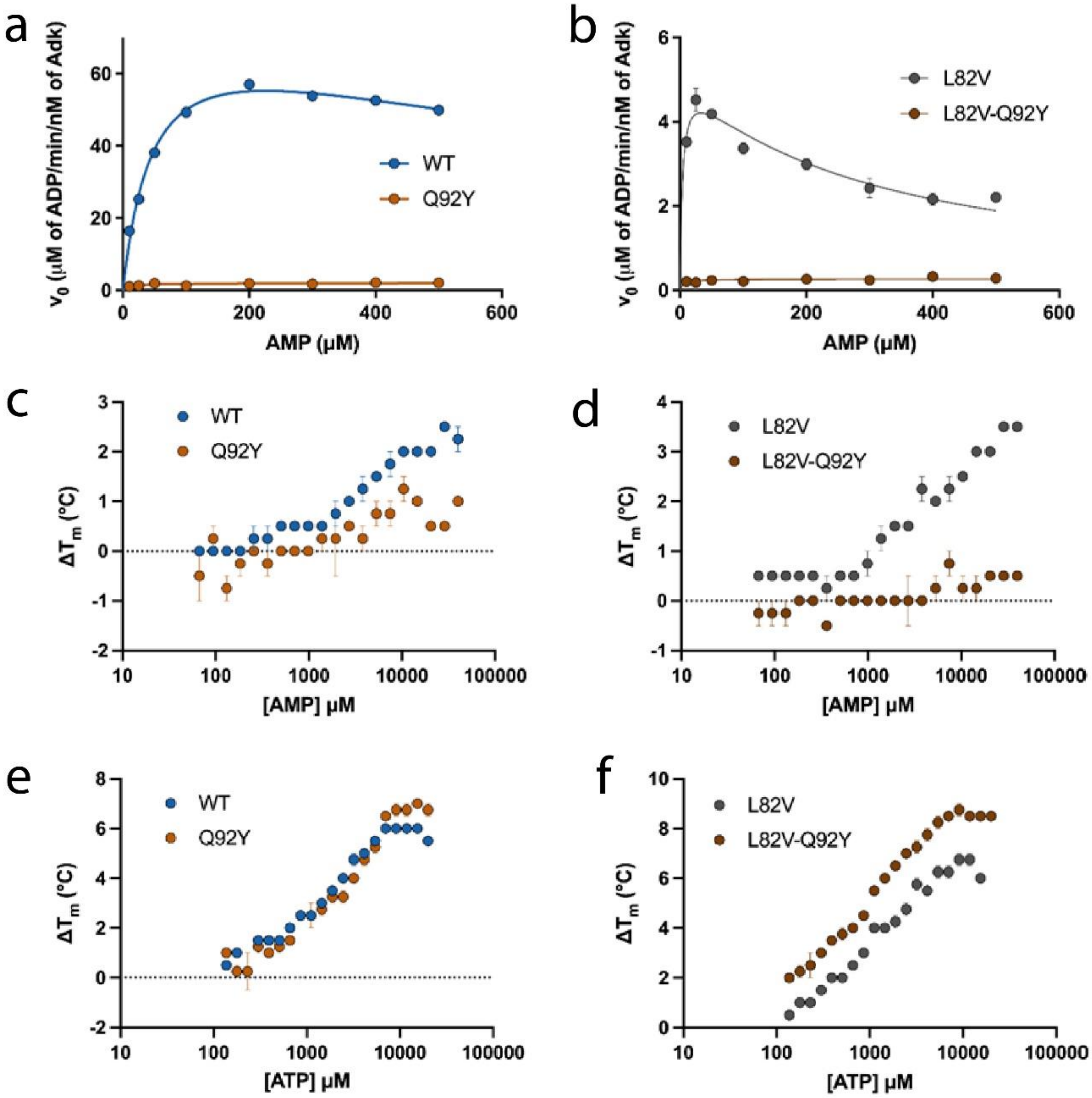

Figure S10: Number of AMP binding sites probed by Differential Scanning Fluorimetry. a-b) Impact of the Q92Y mutation on enzymatic activity for the WT (a) and L82V (b). The Q92Y mutation abolishes crucial interactions between the substrate and the sidechain of Q92 (1), rendering the enzyme inactive. c-d) Titration of WT/WT-Q92Y (c) and L82V/L82V-Q92Y (d) proteins with AMP using Differential Scanning Fluorimetry. The proteins were mixed with different concentrations of AMP and the thermal melting temperatures (T_m) were monitored using the fluorescence of SYPRO Orange. ΔT_m refers to the change in melting temperatures with respect to the unliganded protein. The Q92Y mutation prevents AMP binding. e-f) Titration of WT/WT-Q92Y (e) and L82V/L82V-Q92Y (f) proteins with ATP. The ability to bind ATP is not affected by the Q92Y mutation.

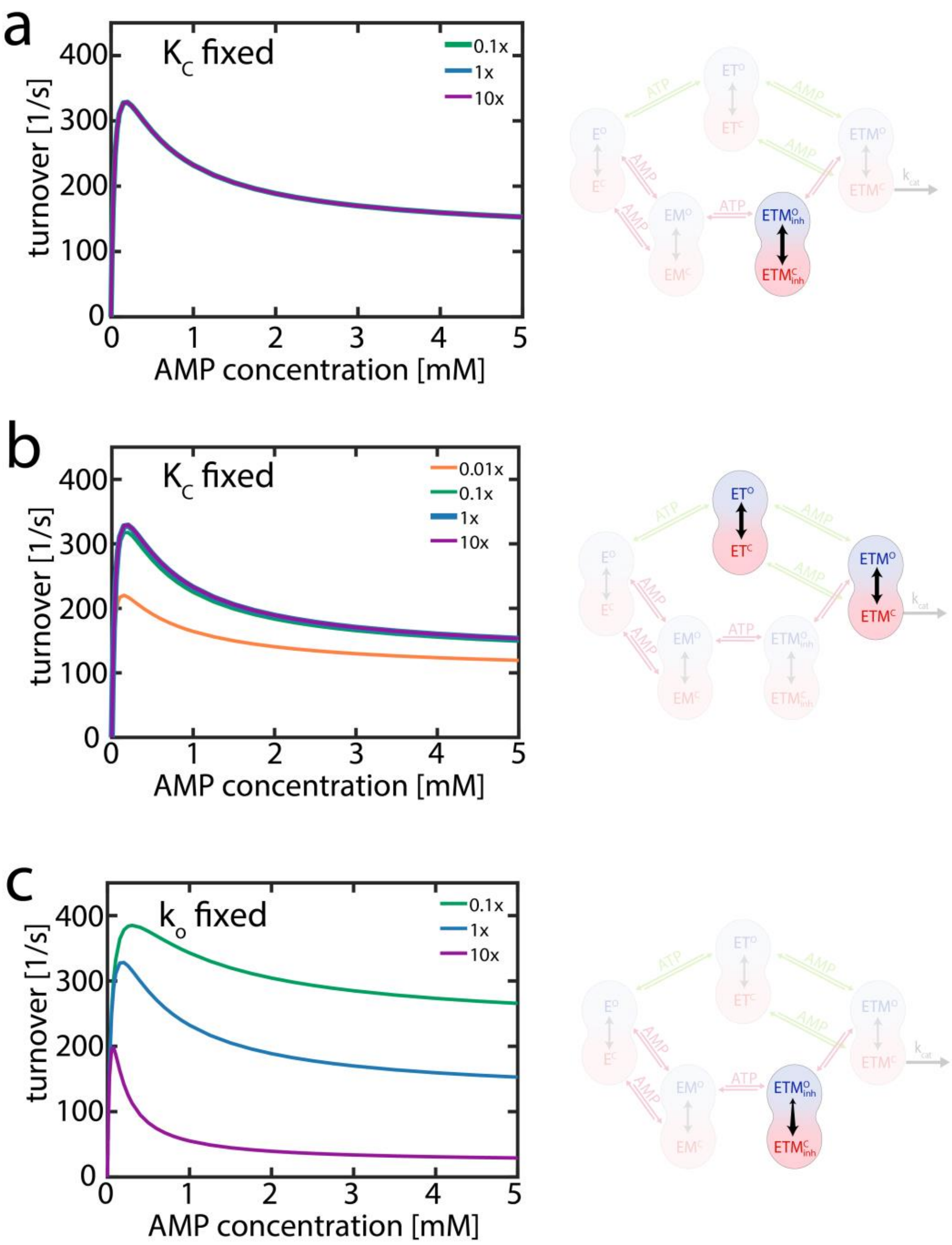

a
K_C fixed
0.1x
1x
10x
turnover [1/s]
AMP concentration [mM]
b
K_C fixed
0.01x
0.1x
1x
10x
turnover [1/s]
AMP concentration [mM]
c
k_o fixed
0.1x
1x
10x
turnover [1/s]
AMP concentration [mM]
ATP
AMP
k_{cat}

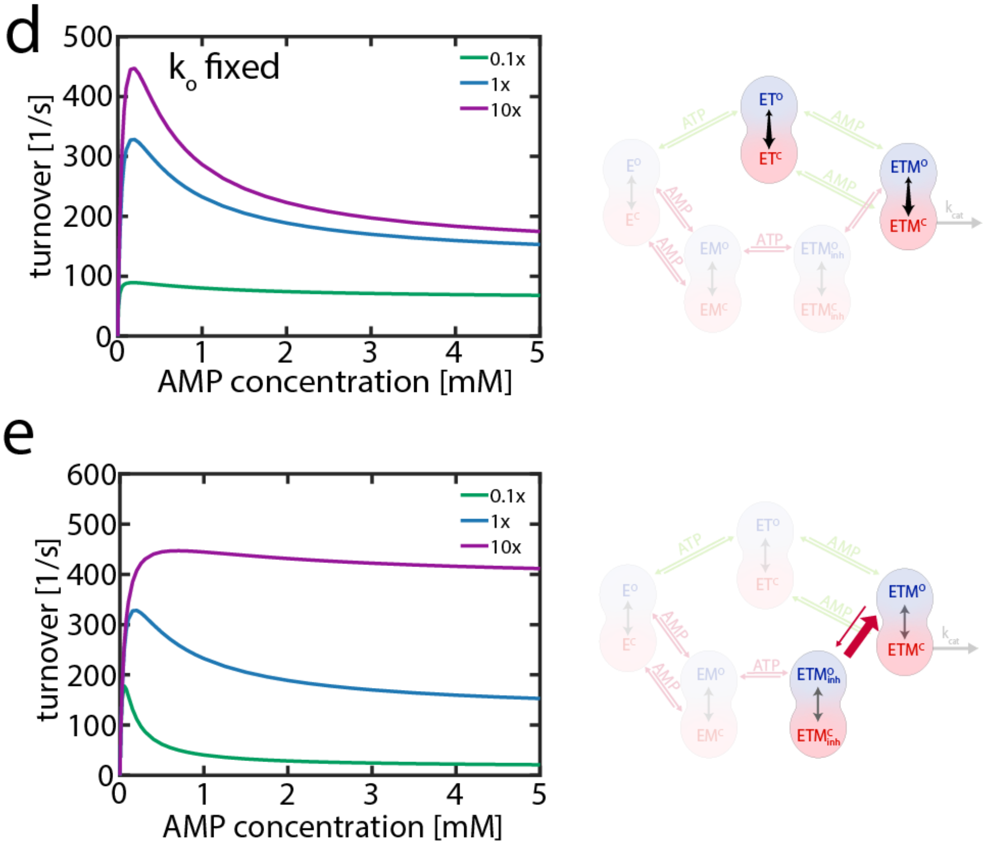

Figure S11: Effect of protein dynamics on simulations of enzymatic activity. The role of conformational dynamics in enzymatic activity was assessed further by altering the rates for specific processes within the conformational cycle (Figure 5). In the left panel, the experimental rates (blue) were scaled by a factor of 0.01 (orange), 0.1 (green) or 10 (purple). The right panel visualizes which rates have been altered. (a) Changing the rate constant for the unproductive closing ($ETM^{O}_{inh} \rightarrow ETM^{C}_{inh}$) has no impact on activity when the conformational equilibrium between open and closed state (K_C) is maintained, i.e. the opening rate is scaled by the same factor. (b) Changing the rate constant for productive closing processes ($ETM^{O} \rightarrow ETM^{C}$ and $ET^{O} \rightarrow ET^{C}$) also has a small effect when K_C is preserved. The turnover is reduced when this step becomes rate limiting. In the orange curve the closing rate is reduced to 270 s^{-1}, i.e. slower than substrate binding (Table S9) and the phosphotransfer step (500 s^{-1} in our model). (c) Changing the rate constant for unproductive closing without preserving the equilibrium does affect both the maximum turnover and substrate inhibition, with stronger inhibition for a faster closing rates. (d) In contrast, for the productive closing states accelerating domain closure has a positive effect on turnover. (e) Variation of the $ETM^{O}_{inh} \rightarrow ETM^{O}$ conversion rate. In the ATP and AMP-dependent simulations (Fig. 5b+c, Fig. S11a-d), a rate of 250 s^{-1} was presumed (blue). Increasing this rate by a factor of 10 (purple) relieves substrate inhibition largely, while a slower rate (0.1x, green) increases substrate inhibition.

Table S1: Kinetic parameters for the purified proteins in the high-throughput study.[a]

Mutation	K_I (μM)[a]	K_M (μM)[a]	ConSurf grades[b]	Frequency of WT amino acid[c] [%]
F19V	($1.2 \cdot 10^5$)	22.1	1	14
I26D	649	2.9	6	49
I26V	560	42.2	6	49
P27S	159	22.5	6	51
P27V	859	33.3	6	51
I29E	906	54.2	8	60
I29M	($1.7 \cdot 10^6$)	46.9	8	60
A37V	412	62.6	4	46
G42F	1024	18.9	2	64
L45S	1437	19.8	6	63
G46E	1799	46	9	99
G46S	1577	35	9	99
A66L	398	27.5	1	15
A66Y	461	14	1	15
K69E	450	22.5	3	37
E70A	421	4.8	5	54
E70S	300	21	5	54
A73E	568	18.9	1	21
N79V	564	16.1	2	24
L83F	1472	29.9	8	75
L83T	($3.3 \cdot 10^6$)	17.8	8	75
L83Y	581	25.6	8	75
I90S	528	159.6	3	29
I90T	(2760)	30.1	3	29
I90V	705	24.2	3	29
I90Y	1592	42.6	3	29
P91E	($3.4 \cdot 10^6$)	141.9	2	31
P91I	419	14.2	2	31
A93L	($2.7 \cdot 10^6$)	95	9	99
A93T	(2010)	23.6	9	99
A93V	1448	50.5	9	99
A95V	689	52.5	7	67
M96I	275	9.3	8	9
E98K	1755	24.1	2	38
E98L	1563	23.5	2	38
E98N	660	35.2	2	38
E98V	375	23.7	2	38
A99E	384	24.2	2	13

G100M	255	15.1	1	47
G100T	259	19.8	1	47
G100V	387	13	1	47
I101A	860	36.4	1	17
I101V	370	8.1	1	17
N102D	233	44.9	1	7
V103M	724	66.6	4	12
Y105E	305	3.9	1	11
Y105T	977	18.3	1	11
Y105V	1067	13.9	1	11
V106F	($4.0\cdot10^6$)	75.7	8	81
V106S	625	21.9	8	81
V117E	1456	7	5	43
A127K	1545	138.3	3	11
A127L	(2304)	41	3	11
A127S	743	51.6	3	11
P128I	200	21.5	1	3
P128T	150	27.1	1	3
K141D	1209	15.4	4	45
V142M	484	22.1	1	28
G144D	584	49.4	5	70
V148D	602	85.5	1	27
V148T	728	30.2	1	27
L153F	($3.3\cdot10^6$)	24.9	8	82
L153T	1729	16.4	8	82
Q173M	489	32.6	3	9
A176Y	223	13.4	2	25
P177A	632	81.1	7	82
P177E	($2.1\cdot10^6$)	66.9	7	82
L178M	($3.5\cdot10^6$)	115.1	7	72
I179N	661	12.1	5	38
I179R	1369	38.3	5	38
G180S	203	27.5	2	13
K184S	1276	48.9	1	23
K184T	290	24	1	23
E187V	560	36.4	1	8
K192T	545	88.7	2	43
A207S	1526	14.9	2	32
A207T	475	21.5	2	32
A207V	1107	26.3	2	32
L209I	330	7	6	24

L209S	331	8.3	6	24
L209T	1762	5.5	6	24
I212A	852	34.1	5	44
I212L	785	25.1	5	44
I212T	823	36.2	5	44
I212V	824	32.6	5	44
L213S	293	43.8	8	93

[a] K_M and K_I were derived by fits to Eq.1. Positions with mutants that result in a K_I <=500 µM for AMP are marked in yellow, while those with K_I >=1500 are marked in green. Magenta refers to positions where both inhibited and uninhibited mutations are observed. Mutants with a very high value in K_I (>=2000) refer to non-inhibited proteins.

[b] ConSurf grades are conservation score derived based on a multiple sequence alignment (MSA) of 150 AK candidates using the ConSurf webserver. Higher numerical values correspond to higher conservation

[c] Frequency of the WT amino acid in the MSA

Table S2: Kinetic parameters for the activity of AK WT in dependence of ATP concentration (Fig. S3a).[a]

ATP [M]	v_{max} [s^{-1}]	K_M [µM]	K_I [µM]
**1**	487(±41)	40.0(±10.6)	2125(±496)
**2**	499(±34)	38.0(±8.7)	2932(±607)
**3**	544(±35)	44.3(±9.2)	3065(±596)
**4**	526(±22)	42.0(±6.2)	4264(±627)
**5**	586(±32)	58.8(±10.4)	4782(±909)

[a] Fits are done according to a model of substrate inhibition involving dead-end complexes (Eq. 1)

Table S3: Comparison of transition rates obtained by H^2MM vs. dwell-time analysis (DTA) for the WT protein.

c_{ATP} [µM]	closing rate[a] [s^{-1}]		opening rate[a] [s^{-1}]	
	H^2MM	DTA	H^2MM	DTA
**0**	527 (±73)	2009[b] (±533)	5851 (±2307)	5225 (±1713)
**5**	1331 (±281)	3049[b] (±615)	7228 (±2803)	7232 (±3055)
**25**	2126 (±239)	2459 (±653)	9159 (±602)	9007 (±553)
**100**	4936 (±1018)	5154 (±1064)	11383 (±1926)	11147 (±1511)
**500**	19569 (±3220)	18213 (±214)	19630 (±1721)	17996 (±630)
**1000**	24856 (±1721)	24678 (±1752)	20058 (±1621)	19097 (±2528)

[a] number in brackets indicate the standard error of the mean of at least two measurements
[b] dwell times extend the length of individual bursts (~250 µs given the chosen burst selection criteria), preventing a reliable analysis

Table S4: Parameters for fitted FCS curves of Figure S8 compared to the parameters obtained from the H^2MM analysis.

	ATP [μM]	0	100	300	1000
FCS results [a]	N	1.59±0.01	1.62±0.01	1.66±0.01	1.68±0.01
	p	0.080±0.002			
	T [μs]	2.7±0.2			
	K	0.069±0.003	0.099±0.003	0.118±0.003	0.146±0.002
	τ_c [μs]	122±2	96±8	78±5	58±3
τ_c [μs] predicted based on H^2MM results [b]		157±12	60±8	32±3	22±1

[a] Error bars represent the standard error of the fit

[b] Values are calculated based on the mean and the standard error of the mean of at least two measurements (τ_c=1/(k_{op} + k_{cl}))

Table S5: Substrate concentrations used in smFRET experiments.

c_{ATP} fixed at 1 mM			c_{AMP} fixed at 1 mM		
**AMP [µM]**	**ADP [µM]**	**Total conc.[a] [µM]**	**ATP [µM]**	**ADP [µM]**	**Total conc.[a] [µM]**
**1**	6.2	1006	**$1*10^{-6}$**	$2*10^{-3}$	$2*10^{-3}$
**2.5**	9.9	1009	**$1*10^{-5}$**	0.02	0.02
**5**	13.9	1014	**$7.5*10^{-5}$**	0.05	0.05
**10**	19.7	1017	**$1*10^{-3}$**	0.2	0.2
**25**	31	1031	**0.01**	0.625	0.635
**50**	44	1044	**0.025**	1	1
**100**	62	1062	**0.1**	1.7	1.8
**250**	95	1095	**0.5**	3.7	4.2
**400**	123	1123	**1**	5.7	6.7
**500**	137	1137	**2.5**	8.4	10.9
**750**	150	1150	**5**	11.8	16.8
**1000**	160	1160	**10**	16.7	26.7
**2000**	230	1230	**35**	31	66
**3000**	327	1327	**50**	37.3	87.3
**5000**	417	1417	**150**	75	225
**10000**	576	1576	**250**	83	333
			**1000**	160	1160
			**2000**	230	2230
			**5000**	417	5417
			**10000**	576	10576

[a] total concentration of substrates that can bind to the LID domain, i.e. ATP and ADP

Table S6: Fitted parameters for the ATP-dependent closure (Figure 3).[a]

	WT		L107I		L82V		F86W	
**AMP**	+	-	+	-	+	-	+	-
$C_{50,ATP}$ **[μM]**	176 (±17)	9.0 (±0.9)	127 (±32)	6.9 (±0.8)	20 (±2.3)	0.55 (±0.04)	223 (±24)	172 (±49)
Occ_{EX} **[%]**	12.8 (±1.0)	15.6 (±1.0)	13.0 (±1.7)	15.3 (±1.0)	15.1 (±1.2)	21.6 (±0.7)	9.9 (±1.0)	11.5 (±2.0)
Occ_{EXT} **[%]**	62.5 (±1.1)	57.1 (±0.6)	56.9 (±1.8)	64.2 (±0.9)	78.0 (±1.1)	78.4 (±0.7)	65.3 (±1.4)	55.8 (±2.3)

[a] Columns/mutants are color-coded according to color-palette used in the main text

Table S7: Parameters for the impact of AMP on LID domain dynamics (Figure 4a-c) according to the model described in Supplementary Note 1.

	WT	L107I	L82V	F86W[a]
$C_{50,AMP}$ **[μM]**	2568 (±820)	422 (±219)	503 (±274)	-
$k_{c,non}$ **[s^{-1}]**	27307 (±752)	29076 (±1266)	15682 (±578)	20047 (±2563)
$k_{c,inh}$ **[s^{-1}]**	50289 (±2795)	48504 (±2731)	24182 (±1264)	-
k_o **[s^{-1}]**	23937 (±1123)[a]	25325 (±1840)[a]	5817 (±553)[a]	21710 (±2924)

[a] No significant change with AMP concentration. Values reflect the average and standard error of the mean of 3 repeated measurements

Table S8: Opening and closing rates used in the simulation of experimental activity patterns.

		WT		L107I		L82V		F86W	
		O→C	C→O	O→C	C→O	O→C	C→O	O→C	C→O
**rate constants [x10^3 s^{-1}]**	**E**	0.5	6	2.2	17	1.5	10	0.34	3.9
	**EM**	2.6	13	7.5	38	16	37	0.36	3.6
	**ET** [a]	27	24	30	25	16	6	20	22
	**ETM$_{inh}$**	50	24	49	25	24	6	20	22
	**ETM** [a]	27	24	30	25	16	6	20	22

[a]The single molecule experiments cannot distinguish between ET and ETM.

Table S9: Nucleotide binding rates used in the simulation of experimental activity patterns.

	WT		L107I		L82V		F86W	
**productive binding rates [μM^{-1} s^{-1}]**	ATP	AMP	ATP	AMP	ATP	AMP	ATP	AMP
	9.31	5.24[a]	12.49	6.29[a]	3.62	13.13[a]	0.68	0.07

[a]In the case of substrate inhibition (WT, L107I and L82V with AMP), parameters were approximated using Eq. (1).

Table S10: Data collection and refinement statistics for the crystal structure of AK L107I in complex with Ap_5A.

Data Collection		
PDB		8BQF
Space group		$P22_12_1$
Cell dimensions	a,b,c (Å)	77.97, 84.49, 218.49
	α,β,γ (˚)	90, 90, 90
No. of copies in a.u.		6
Resolution (Å)		22.03-2.05
Upper resolution shell (Å)		2.12-2.05
Unique reflections		91,211 (8,992)
Completeness (%)		99.69 (100.00)
Average I/σ(I)		20.47 (4.39)
R-pim		0.03909 (0.1585)
CC1/2		0.993 (0.902)
**Refinement**		
Resolution range (Å)		22.03-2.05
No. of reflections (I/σ(I) > 0)		91,091
No. of reflections in test set		4,492
R-working / R-free		0.2183 / 0.2242
No. of protein atoms		9492
No. of water molecules		223
No. of ligand atoms		358
Overall average B factor ($Å^2$)		29.80
Root mean square deviations:		
- bond length (Å)		0.082
- bond angle (°)		3.26
**Ramachandran Plot**		
Most favored (%)		97.60
Additionally allowed (%)		2.08
Outliers (%)		0.32

References

1. C. W. Müller, G. E. Schulz, Structure of the complex between adenylate kinase from Escherichia coli and the inhibitor Ap5A refined at 1.9 Å resolution: A model for a catalytic transition state. *Journal of molecular biology* **224**, 159-177 (1992).
2. B. V. Adkar, S. Bhattacharyya, A. I. Gilson, W. Zhang, E. I. Shakhnovich, Substrate inhibition imposes fitness penalty at high protein stability. *PNAS* **116**, 11265-11274 (2019).
3. H. Ashkenazy, E. Erez, E. Martz, T. Pupko, N. Ben-Tal, ConSurf 2010: calculating evolutionary conservation in sequence and structure of proteins and nucleic acids. *Nucleic acids research* **38**, W529-W533 (2010).
4. H. Ashkenazy *et al.*, ConSurf 2016: an improved methodology to estimate and visualize evolutionary conservation in macromolecules. *Nucleic acids research* **44**, W344-W350 (2016).
5. P. Liang, G. N. Phillips Jr., M. Glaser, Assignment of the nucleotide binding sites and the mechanism of substrate inhibition of Escherichia coli adenylate kinase. *Proteins: Structure, Function, and Bioinformatics* **9**, 28-36 (1991).
6. W. Ferdinand, The interpretation of non-hyperbolic rate curves for two-substrate enzymes. A possible mechanism for phosphofructokinase. *Biochemical Journal* **98**, 278-283 (1966).
7. I. H. Segel, *Enzyme Kinetics: Behavior an Analysis of Rapid Equilibrium and Steady-State Enzyme Systems* (John Wiley and Sons Ltd., New York, 1993).
8. P. Evans, Scaling and assessment of data quality. *Acta Crystallographica Section D* **62**, 72-82 (2006).
9. S. French, K. Wilson, On the treatment of negative intensity observations. *Acta Crystallographica Section A: Crystal Physics, Diffraction, Theoretical and General Crystallography* **34**, 517-525 (1978).
10. A. J. McCoy, Solving structures of protein complexes by molecular replacement with Phaser. *Acta Crystallogr D Biol Crystallogr* **63**, 32-41 (2007).
11. G. N. Murshudov, A. A. Vagin, E. J. Dodson, Refinement of macromolecular structures by the maximum-likelihood method. *Acta Crystallographica Section D: Biological Crystallography* **53**, 240-255 (1997).
12. P. V. Afonine *et al.*, Towards automated crystallographic structure refinement with phenix.refine. *Acta Crystallographica Section D: Biological Crystallography* **68**, 352-367 (2012).
13. P. Emsley, K. Cowtan, Coot: model-building tools for molecular graphics. *Acta Crystallographica Section D* **60**, 2126-2132 (2004).
14. H. Y. Aviram *et al.*, Direct observation of ultrafast large-scale dynamics of an enzyme under turnover conditions. *PNAS* **115**, 3243-3248 (2018).
15. X. R. Sheng, X. Li, X. M. Pan, An iso-random Bi Bi mechanism for adenylate kinase. *Journal of Biological Chemistry* **274**, 22238-22242 (1999).
16. S. Kuby, E. Noltman (1962) ATP-Creatine Transphosphorylase. The Enzymes. (Academic Press: New York, NY, USA).
17. M. Pirchi *et al.*, Photon-by-photon hidden Markov model analysis for microsecond single-molecule FRET kinetics. *J. Phys. Chem. B* **120**, 13065-13075 (2016).
18. H. Mazal *et al.*, Tunable microsecond dynamics of an allosteric switch regulate the activity of a AAA+ disaggregation machine. *Nat. Commun.* **10**, 1438 (2019).

19. N. K. Lee *et al.*, Accurate FRET Measurements within Single Diffusing Biomolecules Using Alternating-Laser Excitation. *Biophysical Journal* **88**, 2939-2953 (2005).
20. J. Hohlbein, T. D. Craggs, T. Cordes, Alternating-laser excitation: single-molecule FRET and beyond. *Chemical Society Reviews* **43**, 1156-1171 (2014).
21. B. Hellenkamp *et al.*, Precision and accuracy of single-molecule FRET measurements—a multi-laboratory benchmark study. *Nature Methods* **15**, 669-676 (2018).
22. I. V. Gopich, A. Szabo, Decoding the pattern of photon colors in single-molecule FRET. *The Journal of Physical Chemistry B* **113**, 10965-10973 (2009).
23. M. Pirchi (2013) Mapping the free-energy landscape of multi-domain proteins by single-molecule FRET spectroscopy. (Weizmann Institute of Science, Rehovot, Israel).
24. T. Torres, M. Levitus, Measuring Conformational Dynamics: A New FCS-FRET Approach. *The Journal of Physical Chemistry B* **111**, 7392-7400 (2007).
25. E. S. Price, M. S. DeVore, C. K. Johnson, Detecting Intramolecular Dynamics and Multiple Förster Resonance Energy Transfer States by Fluorescence Correlation Spectroscopy. *The Journal of Physical Chemistry B* **114**, 5895-5902 (2010).
26. M. A. Sinev, E. V. Sineva, V. Ittah, E. Haas, Domain Closure in Adenylate Kinase. *Biochemistry* **35**, 6425-6437 (1996).
27. M. A. Sinev, E. V. Sineva, V. Ittah, E. Haas, Towards a mechanism of AMP-substrate inhibition in adenylate kinase from Escherichia coli. *FEBS Letters* **397**, 273-276 (1996).
28. G. Bonnet, O. Krichevsky, A. Libchaber, Kinetics of conformational fluctuations in DNA hairpin-loops. *Proceedings of the National Academy of Sciences* **95**, 8602-8606 (1998).
